# The impact of patient-facing digital health interventions on patient safety outcomes: a meta-analysis of meta-analyses

**DOI:** 10.1093/oodh/oqag031

**Published:** 2026-08-29

**Authors:** Goran Erfani, Jemma McCready, Beckie Gibson, Alison Steven, John Unsworth, Heidi Siira, Erika Jarva, Kristina Mikkonen, Marco Tomietto

**Affiliations:** School of Healthcare and Nursing Sciences, Faculty of Health and Wellbeing, Northumbria University, Newcastle upon Tyne, NE7 7TR, United Kingdom; Department of Social Work, Education and Community Wellbeing, Faculty of Health and Life Sciences, Northumbria University, Newcastle upon Tyne, NE7 7TR, United Kingdom; Psychology Department, Northumbria University, Newcastle upon Tyne, NE7 7TR, United Kingdom; School of Healthcare and Nursing Sciences, Faculty of Health and Wellbeing, Northumbria University, Newcastle upon Tyne, NE7 7TR, United Kingdom; School of Healthcare and Nursing Sciences, Faculty of Health and Wellbeing, Northumbria University, Newcastle upon Tyne, NE7 7TR, United Kingdom; Research Unit of Health Sciences and Technology, Faculty of Medicine, University of Oulu, P.O.Box 8000FI-90014, Oulu, Finland; Research Unit of Health Sciences and Technology, Faculty of Medicine, University of Oulu, P.O.Box 8000FI-90014, Oulu, Finland; School of Healthcare and Nursing Sciences, Faculty of Health and Wellbeing, Northumbria University, Newcastle upon Tyne, NE7 7TR, United Kingdom; Research Unit of Health Sciences and Technology, Faculty of Medicine, University of Oulu, P.O.Box 8000FI-90014, Oulu, Finland; Medical Research Center Oulu, Oulu University Hospital and University of Oulu, P.O.Box 8000FI-90014, Oulu, Finland; School of Healthcare and Nursing Sciences, Faculty of Health and Wellbeing, Northumbria University, Newcastle upon Tyne, NE7 7TR, United Kingdom; Research Unit of Health Sciences and Technology, Faculty of Medicine, University of Oulu, P.O.Box 8000FI-90014, Oulu, Finland; Centre for Health and Social Equity, Northumbria University, Newcastle upon Tyne, NE7 7TR, United Kingdom

**Keywords:** digital health, healthcare, patient safety, digital solutions, meta-analysis, patient-facing interventions

## Abstract

Patient safety is a critical concern in healthcare, with preventable adverse events contributing significantly to morbidity and mortality. Digital health interventions (DHIs) are increasingly integrated into healthcare systems to improve patient safety outcomes. However, their effectiveness remains unclear. This study focuses on DHIs adopted by patients and, in detail, it aims to assess the effectiveness of patient-facing DHIs in improving patient safety outcomes, including medication adherence, healthcare utilization, falls, mortality, adverse events, relapse rate, and readmissions. This meta-analysis of meta-analyses follows the Preferred Reporting Items for Systematic Reviews and Meta-Analyses guidelines, and it is registered in PROSPERO. The eligible meta-analyses were identified across major databases (PubMed, Web of Science, Cochrane Reviews, EPISTEMONIKOS, CINAHL, ProQuest, Scopus, PsychARTICLES) from 2010 until 2024. The quantitative synthesis adopted a random-effects model. The corrected covered area method quantified the studies’ overlap and heterogeneity was assessed using the I^2^ statistic. Publication bias was examined using Egger’s test. The JBI Critical Appraisal Checklist was adopted. In total, 72 meta-analyses were included. Findings indicate that patient-facing DHIs improve medication adherence (SMD = 0.33; 95% CI = 0.22–0.44), reduce mortality risk (RR = 0.84; 95% CI = 0.78–0.90) and hospital readmissions (RR = 0.90; 95% CI = 0.81–0.98). Additionally, patient-facing DHIs reduce fall risk among older adults (SMD = 0.30; 95% CI = 0.13–0.46). However, the impact on healthcare utilization reported increased patient visits, while relapse rate (RR = 1.00; 95% CI = 0.87–1.16) and adverse events (RR = 0.94; 95% CI = 0.66–1.34) showed no significant association with patient-facing DHIs. While these digital solutions demonstrate the potential to enhance medication adherence, reduce mortality, and prevent falls, their effectiveness varies. Challenges such as usability issues, digital literacy gaps, and infrastructure limitations should be addressed to optimize patient safety outcomes.

## Introduction

Patient safety is defined as the absence of preventable harm to a patient and the reduction of the risk of unnecessary harm associated with health care to an acceptable minimum (World Health Organization 2023). Patient safety incidents are a growing global health concern and one of the leading causes of death and disability worldwide (Lima Júnior et al. 2023). It is estimated that one in 10 patients is harmed in healthcare settings due to unsafe care, and approximately 50% of adverse events in healthcare are preventable among the Organisation for Economic Co-operation and Development (OECD) countries (Slawomirski and Klazinga 2022).

Digital health interventions (DHIs) are considered a promising approach to improve patient safety and are increasingly adopted in healthcare and nursing care. Specifically, DHIs include any health service or treatment delivered using technology that aims to facilitate, capture or exchange knowledge (Murray et al. 2016). DHIs such as electronic health records (EHRs), telemedicine, mobile health (mHealth), wearable devices, and artificial intelligence (AI)-driven solutions are increasingly used to monitor patient data, provide timely alerts, and enable proactive interventions, which in turn may improve patient safety (Ye et al. 2024). In detail, DHIs broadly encompass both non-patient-facing (e.g. EHRs, e-prescribing, clinical decision support) and patient-facing (e.g. telemedicine, mHealth, wearable devices) technologies. Non-patient-facing technologies operate primarily at the organizational and system level, by providing the healthcare organizations and professionals with tools to support clinical decisions and data. On the other hand, patient-facing DHIs hold direct relevance to patient engagement, self-care, and safety outcomes by providing the patients with self-monitoring devices and digital solutions to manage their health (World Health Organization 2018).

These solutions have the potential to minimize human errors, optimize clinical workflows, and empower patients in their health management. Furthermore, the integration of digital technologies into healthcare has the potential to enhance patient safety, efficiency, and care quality. However, realizing these benefits requires effectively implementing these technologies and developing digital competencies among nurses and healthcare professionals (Longhini et al. 2024), alongside empowering patients to properly integrate these solutions into their health management (Jarva et al. 2024).

Digital health has evolved as a core pillar in healthcare systems, driven by advancements in information and communication technologies (ICT). As outlined by the World Health Organization (WHO), digital health encompasses strategies that leverage technology to strengthen health systems, improve access to services, and enhance health outcomes globally (Kulju et al. 2024). Furthermore, it has been demonstrated that the benefits of DHIs are multifaceted, including improved accuracy in clinical documentation, better communication among care teams, enhanced patient engagement, and data-driven decision-making (Jarva et al. 2024, Konttila et al. 2019).

Research proves that patient-facing DHIs, such as telemedicine, can significantly reduce adverse events by improving the accuracy of patient information and enabling real-time monitoring and remote consultations, hence, improving patients’ self-care and empowerment (Jarva et al. 2024, Konttila et al. 2019). For instance, AI algorithms integrated into clinical workflows can predict patient deterioration or assist in diagnostic precision, improving safety outcomes. De Micco et al. 2025 emphasize how predictive analytics driven by machine learning can reduce hospital readmission rates, providing an additional layer of safety for patients. Similarly, Jafleh et al., 2024 highlight the role of wearable devices in facilitating continuous patient monitoring, which helps in early detection of critical events. Yet, the WHO and the European Union highlight that challenges remain in ensuring equitable access, addressing the digital divide, fostering adoption among healthcare providers, and establishing a global strategy on digital health to address these challenges (Jarva et al. 2024, Kulju et al. 2024).

Competencies in digital health are increasingly recognized as essential for nurses and healthcare professionals, as reflected in various international frameworks and strategies. Nursing care is internationally recognized as a major component of patient safety, and effectively integrating both patient-facing DHIs into nursing care can further enhance patient safety outcomes, such as medication adherence, falls prevention, readmissions, and mortality. In this vein, the European Union’s Digital Education Action Plan emphasizes the need for robust digital skills training across all sectors, including healthcare (Jarva et al. 2024). Similarly, national initiatives such as the Finnish Nurses Association’s strategy for digital health services aim to integrate digital health competencies into clinical education and practice (Kulju et al. 2024). These efforts are supported by empirical evidence indicating that targeted educational interventions can significantly improve healthcare professionals’ knowledge, skills, and confidence in using digital tools (Kulju et al. 2024).

A meta-analysis by Li and Chen (2024) further underscores the importance of digital competence in enhancing patient-centered care. Their findings reveal that nurses and healthcare professionals with advanced digital literacy are more likely to promote and adopt patient-facing technologies, such as mobile health apps, thereby facilitating better adherence to treatment plans and improving overall health outcomes.

Despite the promise of DHIs, their effectiveness in improving patient safety is contingent upon overcoming several barriers. Resistance to change, insufficient training, and lack of institutional support (from educational resources to technical infrastructures) are frequently cited as impediments to successful implementation (Kulju et al. 2024). A systematic review identified that healthcare professionals, and nurses in particular, often struggle with inadequate infrastructure, time constraints, and limited digital literacy, which can hinder the effective use of technologies like EHRs and telehealth platforms (Kulju et al. 2024).

Additionally, the usability of digital tools plays a critical role in their adoption. Poorly designed systems that do not align with clinical workflows can increase the cognitive load on healthcare providers, leading to errors and reduced efficiency, as unintended consequences. Addressing these challenges requires a comprehensive approach, combining technical refinements with organizational and collegial support from nursing leaders and hospital administrators (Jarva et al. 2024, Kulju et al. 2024).

Furthermore, De Micco et al. 2025 identify systemic issues, such as interoperability challenges between different digital platforms, as significant obstacles. Other studies (Jafleh et al., 2024) echo this concern, highlighting the need for standardized systems that facilitate seamless data exchange while maintaining data security and privacy.

The relationship between digital health and patient safety is complex and dynamic. While the adoption of DHIs can reduce medication errors, improve diagnostic accuracy, and enhance care coordination, the potential risks associated with technology misuse or system failures might affect these outcomes. For instance, cybersecurity breaches, data inaccuracies, and over-reliance on automated decision-making tools can compromise patient safety if not managed appropriately. These risks can be mitigated by enhancing the healthcare professionals’ and patients’ digital competences and by providing adequate organizational and educational support when introducing new digital solutions in the healthcare settings (Kulju et al. 2024, De Micco et al. 2025).

While DHIs represent a broad category, this study specifically focuses on patient-facing DHIs, as they are more directly linked to the patients’ experience and they involve the active integration of patients, healthcare organizations, and healthcare professionals. Moreover, a growing body of systematic reviews shows that patient-facing DHIs are positively associated with patient safety outcomes such as readmissions (Grygorian et al. 2024) and medication adherence (Vitija et al. 2022); however, the effects remain inconsistent. It is also unclear how effective patient-facing DHIs are across different healthcare settings and populations of patients, spanning from hospital to community settings, and from acute to chronic conditions across different age categories. As there are several published systematic reviews with meta-analyses, a quantitative synthesis of existing meta-analyses is appropriate to provide a comprehensive overview of the effects of DHIs on patient safety (Biondi 2016). The effectiveness of patient-facing DHIs in improving patient safety outcomes needs to be systematically explored as the interplay of technology, human factors, and organizational support are complex and can affect the actual effectiveness of these interventions on patient safety outcomes. Therefore, this meta-analysis of meta-analyses addresses this gap and aims to provide the first comprehensive quantitative synthesis of meta-analyses to explore the effectiveness of patient-facing DHIs in improving patient safety outcomes.

## Materials and methods

### Objective

This meta-analysis of meta-analyses (meta-meta-analysis) aimed to evaluate the effectiveness of patient-facing digital health interventions (DHIs) in enhancing patient safety outcomes, based on the existing literature. The Preferred Reporting Items for Systematic Reviews and Meta-Analyses (PRISMA) guidelines (Page et al. 2021) have been adopted.

### Protocol and registration

The review protocol was pre-registered with the International Prospective Register of Systematic Reviews (PROSPERO, registration number: CRD42024528569). Deviations from the registered protocol are detailed in the section ‘Deviations from the Registered Protocol’, with justifications provided. Searches of PROSPERO, the Joanna Briggs Institute Registries, and the Open Science Framework confirmed the absence of similar pre-existing protocols.

### Eligibility criteria

The inclusion criteria followed the PICOS framework (Population, Intervention, Comparator/Context, Outcome, Study type/Setting) (Richardson et al., 1995). In detail, studies including participants accessing healthcare services, regardless of age, gender, ethnicity and health condition under investigation were included (P). Regarding the interventions (I), digital health interventions that utilize patient-facing eHealth technologies (e.g. telemedicine) or mHealth technologies (e.g. smartphones, mobile sensors) were included, and non-patient-facing technologies (e.g. EHRs, e-prescribing, clinical decision supports) were excluded. Additionally, studies using eHealth or mHealth technologies for public health interventions (e.g. health promotion, health prevention, epidemiology), preventative behavior change (e.g. reducing smoking, alcohol consumption), self-management (e.g. users self-manage a specific condition through health behaviors such as exercise management), and diagnosis (e.g. supporting diagnosis of a specific condition) were excluded unless they also included a patient safety outcome of interest (e.g. medication adherence). Regarding context (C) research conducted in any country and all healthcare settings (e.g. acute care, healthcare settings, outpatient settings, community settings) was included. All types of comparator groups were considered, with no exclusion criteria based on comparator type. The main outcomes (O) of this study were any variables that are related to patient safety: hospital admission rates, readmission rates, mortality rates, relapse rates, adverse side-effects, medication safety (e.g. medication errors, adverse drug events), patient engagement (e.g. treatment adherence, medication adherence), and patient monitoring (e.g. detection of deterioration/complications, risk factors, clinical outcomes) (De Micco et al. 2025). Studies that evaluate the cost-effectiveness of digital health interventions were excluded.

Regarding the study type (S), only systematic reviews with meta-analyses were included and, specifically, they were required to meet at least two of the following criteria:

1. Pre-specified eligibility criteria (e.g. registration in PROSPERO or an established protocol).
2. Use of systematic methods that were explicit and reproducible (e.g. PRISMA).
3. Inclusion of risk of bias or quality assessment ensured by two or more independent reviewers.

Meta-analyses that included studies outside the healthcare settings were included only if relevant findings from healthcare settings were explicitly reported. Conference abstracts were excluded due to insufficient detail for full data extraction (Hackenbroich et al. 2022).

### Search strategy

Scoping searches were conducted to develop a preliminary search strategy, which was peer-reviewed by a librarian specialist at Northumbria University using the Peer Review of Electronic Search Strategies (PRESS) checklist (McGowan et al. 2016). Searches were conducted in databases such as PubMed, Web of Science, Cochrane Reviews, EPISTEMONIKOS, CINAHL, ProQuest, Scopus, PsychARTICLES, limited to publications from 2010 until June 2024, and restricted to English-language reviews. The timeframe of the search strategy starting from 2010 was based on evidence supporting that this period marks a notable acceleration in mHealth research (Cameron et al. 2015). The search strategy is described in Table 1 and it was adapted to the requirements of individual databases. Forward and backward citation searches, as well as recommendations from colleagues, were used to supplement the database search. Grey literature was identified through hand-searching, OpenGrey, Google Scholar, and preprint servers like MedRxiv. For instance, searches on Google Scholar were conducted using key terms from our strategy (e.g. digital health, meta-analysis). Given the large volume of results, screening was focused on the first 250 results sorted by relevance for each search string, as this platform’s ranking algorithm prioritizes potentially relevant studies (Haddaway et al. 2015).

**Table 1:** Search string for assessing the effectiveness of digital health interventions on patient safety outcomes.

Intervention	Outcome	Study type
‘digital health’ OR mHealth OR eHealth OR telemedicine OR telerehabilitation OR ‘health information technology’ OR ‘mobile health’ OR telehealth OR ‘virtual health’ OR ‘remote health’ OR ‘artificial intelligence’ OR AI NOT clinical decision support OR e-prescribing OR ‘patient electronic portals’ OR ‘electronic medical records’	AND ‘patient safety’ OR ‘adverse health care event’ OR ‘adverse drug event’ OR health care errors OR ‘medication errors’ OR ‘patient mortality’ OR ‘adverse effect’ OR treatment adherence OR ‘medication adherence’ OR ‘admission rates’ OR ‘readmission rates’ OR treatment compliance OR falls	AND ‘systematic review’ OR ‘meta-analysis’

### Deviations from the protocol

While the initial eligibility criteria specified meta-analyses published from 2010 onwards (Cameron et al. 2015), as declared in the previous section, the search strategy also incorporated forward and backward citation searching and recommendations from colleagues. Therefore, through backward citation searching, two additional meta-analyses published before 2010 (Clark et al. 2007, Klersy et al. 2009) were identified as meeting all other eligibility criteria in terms of population, intervention, and outcome, and were included in accordance with the supplementary search methods. Moreover, patient-facing DHIs such as telemonitoring and telemedicine in heart failure populations represent an area of research that was already well-established prior to 2010.

Additionally, the original protocol was designed to also conduct a qualitative synthesis of the results. However, given the considerable amount of studies retrieved, it was decided to focus on the quantitative synthesis, as it was considered more informative in synthesizing complex evidence.

### Selection of studies

**Figure 1:**
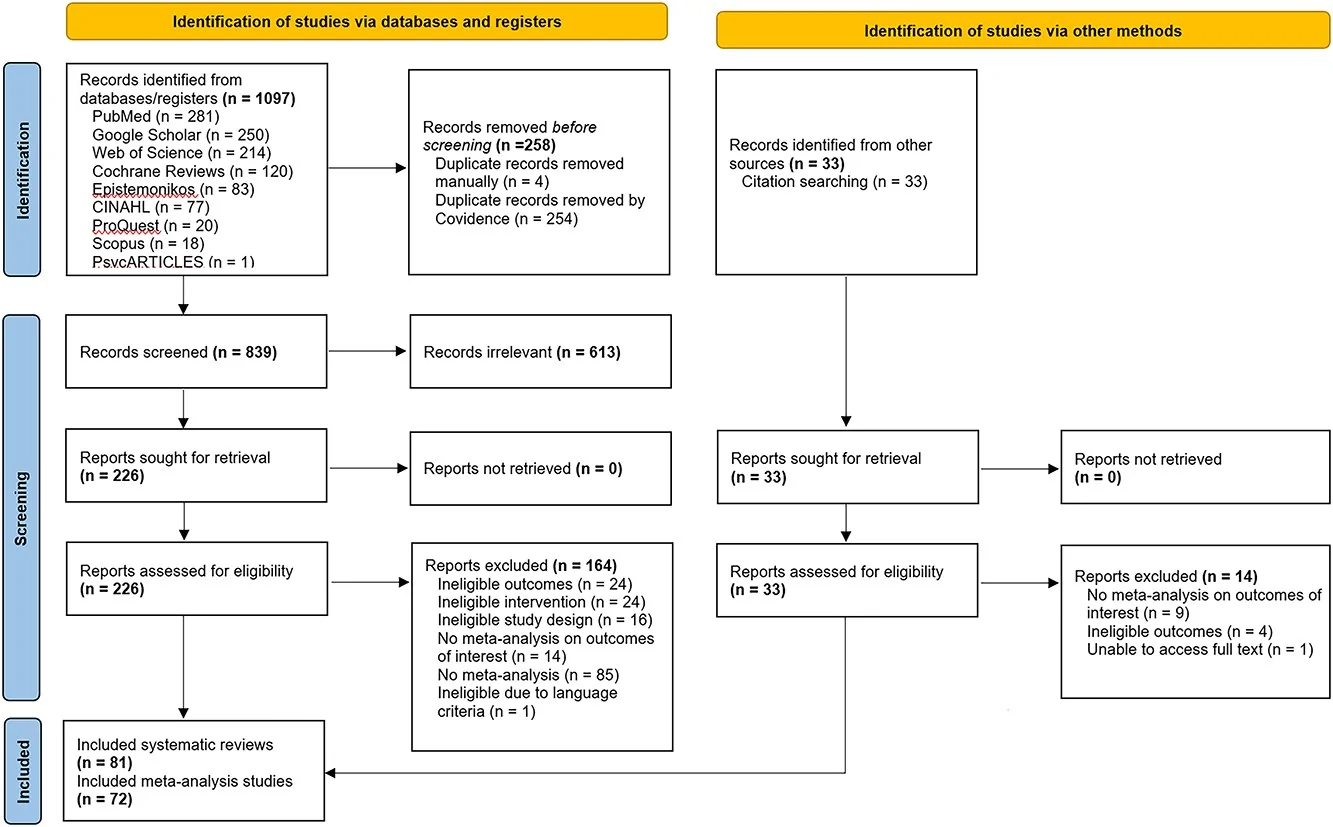
PRISMA diagram.

Search results were exported to Endnote for duplicate removal and subsequently uploaded to Covidence, a systematic review management tool (Covidence 2023). Two independent reviewers screened titles and abstracts, followed by full-text reviews, based on predefined eligibility criteria. Discrepancies were resolved through discussion or consultation with a third reviewer, as required. The screening process is described in the PRISMA diagram (Fig. 1).

### Data extraction and quality assessment

Data extraction followed Cochrane guidelines (Pollock et al. 2020) and included details about the meta-analyses, characteristics of included primary studies, meta-analytic results, and quality assessments (Table S1—Appendix I summarizes the key characteristics of the included reviews and their quality appraisal). The data extraction form was pilot tested within the research team and completed independently by two reviewers, with disagreements resolved through discussion.

The quality of the included reviews was assessed using the JBI Critical Appraisal Checklist (Aromataris et al. 2015, Joanna Briggs Institute 2017). This approach was preferred compared to the Cochrane risk of bias assessment because it considers not only risks of bias but also aspects of methodological quality and relevance (Aromataris et al. 2015, Joanna Briggs Institute 2017, Pieper et al. 2014). The checklist evaluated criteria such as clarity of research questions, protocol registration, inclusion criteria, search strategy comprehensiveness, independent study selection, and evidence synthesis. Quality scores informed confidence in the included reviews, following previously applied scoring criteria (Pieper et al. 2014) (Table S1—Appendix I).

The data extraction process was conducted using Microsoft Excel. Data from meta-analyses were categorized and extracted separately as continuous and dichotomous variables. For continuous variables, the extracted information included the author names, publication years, types of interventions, assessed outcomes, model types (random or fixed effects), sample characteristics, the number of studies and participants, standardized mean difference (SMD) or mean difference (MD), confidence intervals (CIs), *P*-values, I-squared values, and heterogeneity *P*-values. For dichotomous variables, the extracted data encompassed similar details, with the addition of event occurrences and total sample sizes for both experimental and control groups where applicable. In instances where sample sizes were not explicitly reported, they were estimated based on the available information from the primary studies. In rare instances, primary studies were consulted due to unclear information in meta-analyses. Furthermore, if necessary, any missing data were requested from the authors of the meta-analyses.

### Strategy for data synthesis

The corrected covered area (CCA) method quantified overlap among primary studies, categorized into slight (0%–5%), moderate (6%–10%), high (11%–15%), or very high (>15%) overlap (Pieper et al. 2014).

Meta-meta-analysis integrates results from multiple meta-analyses to achieve more precise and robust conclusions with increased statistical power afforded by the larger accumulated sample size (Cleophas et al. 2017, Singh et al. 2024). In this study, meta-analytic pooling was conducted for outcomes reported quantitatively [as risk ratios, odds ratios (ORs), or SMDs] by at least two eligible meta-analyses. Specifically, a meta-meta-analysis of the outcomes of interest was conducted by pooling the effect sizes and 95% CIs from each individual meta-analysis. A profile likelihood random effects model was used to estimate the overall effect and account for variations among studies (Veroniki et al. 2019). This meta-analysis of meta-analyses was designed to provide a comprehensive overview of the effectiveness of patient-facing DHIs on patient safety outcomes. Therefore, performing a subgroup analysis by intervention subtype or patient population was not considered beneficial to the comprehensiveness of this study. This methodological choice is consistent with established umbrella-review methodology (Biondi 2016), which recommends that reviews of reviews prioritize breadth of synthesis across an outcome domain over granular stratification by intervention characteristics, particularly when the underlying evidence base is itself composed of aggregated reviews rather than primary studies.

For outcomes measured as continuous variables, all unstandardized MD outcomes were converted to SMD using pooled standard deviation (SD) (Deeks et al. 2019). The summarized effect sizes (SMDs) were presented along with their corresponding 95% CIs through forest plots. If multiple SMD effect sizes were reported for different types of digital health interventions for one outcome in a study, we calculated the aggregated SMD with a 95% CI to avoid overestimation in meta-analyses (Deeks et al. 2019).

For dichotomous variables, OR estimates were converted into a scale that aligns with risk ratio (RR) by using a square-root approximation method that does not depend on the rare-outcome assumption (VanderWeele 2017). The square-root transformation was chosen over reporting the OR directly because it reduces this bias without requiring the rare-outcome assumption on which the conventional practice of treating OR as approximately equal to RR depends. In detail, this assumption could not be reliably verified across the heterogeneous outcome prevalences reported by the included meta-analyses. Next, both RRs and converted ORs were log-transformed, and standard errors (SEs) from 95% CIs were estimated in accordance with Deeks et al. (2019). This approach was preferred as it is widely recognized and adopted in meta-epidemiological studies as a pragmatic way of approximating risk ratios from ORs when only limited information is available. Moreover, in our data, the baseline risks generally fell within a moderate range where this approach can handle properly any possible biases (Deeks et al. 2019).

The I^2^ statistic was used to quantify the degree of heterogeneity in the included studies. According to Cochrane standards, heterogeneity is considered low if I^2^ = 0%–25%, moderate if I^2^ ≥ 25%–50%, substantial if I^2^ ≥ 50%–75%, and high if I^2^ ≥ 75%–100% (Deeks et al. 2019). The magnitude of effect size was classified as follows: very small (<0.20), small (between 0.20 to 0.50), medium (between 0.50 to 0.80) and large (greater than 0.80) (Cohen, 1988). Risk of publication bias was assessed using Egger’s test when >10 studies were included in the meta-meta-analysis for the outcome in consideration (Deeks et al. 2019, Egger et al. 1997). Funnel plots were also generated for the included studies and presented in Supplementary Appendix II (from Figs S1 to S10). A *P*-value of <.05 was considered statistically significant for all analyses. Clinical significance was evaluated by comparing meta-analysis effect sizes with the Minimally Important Difference (MID) (Johnston et al. 2013). The analyses were performed using IBM SPSS Statistics version 28 (IBM Corp 2021).

## Results

This section presents the results of the meta-meta-analyses selected according to the PRISMA guidelines. Figure 1 illustrates the PRISMA flow diagram of the study selection process. The overlap between the studies included in this meta-meta-analysis was 0.73% (CCA), indicating a slight overlap. To aid interpretation, included outcomes were additionally mapped onto Donabedian’s Structure–Process–Outcome framework (Table S3—Appendix IV).

### Medication adherence

**Figure 2:**
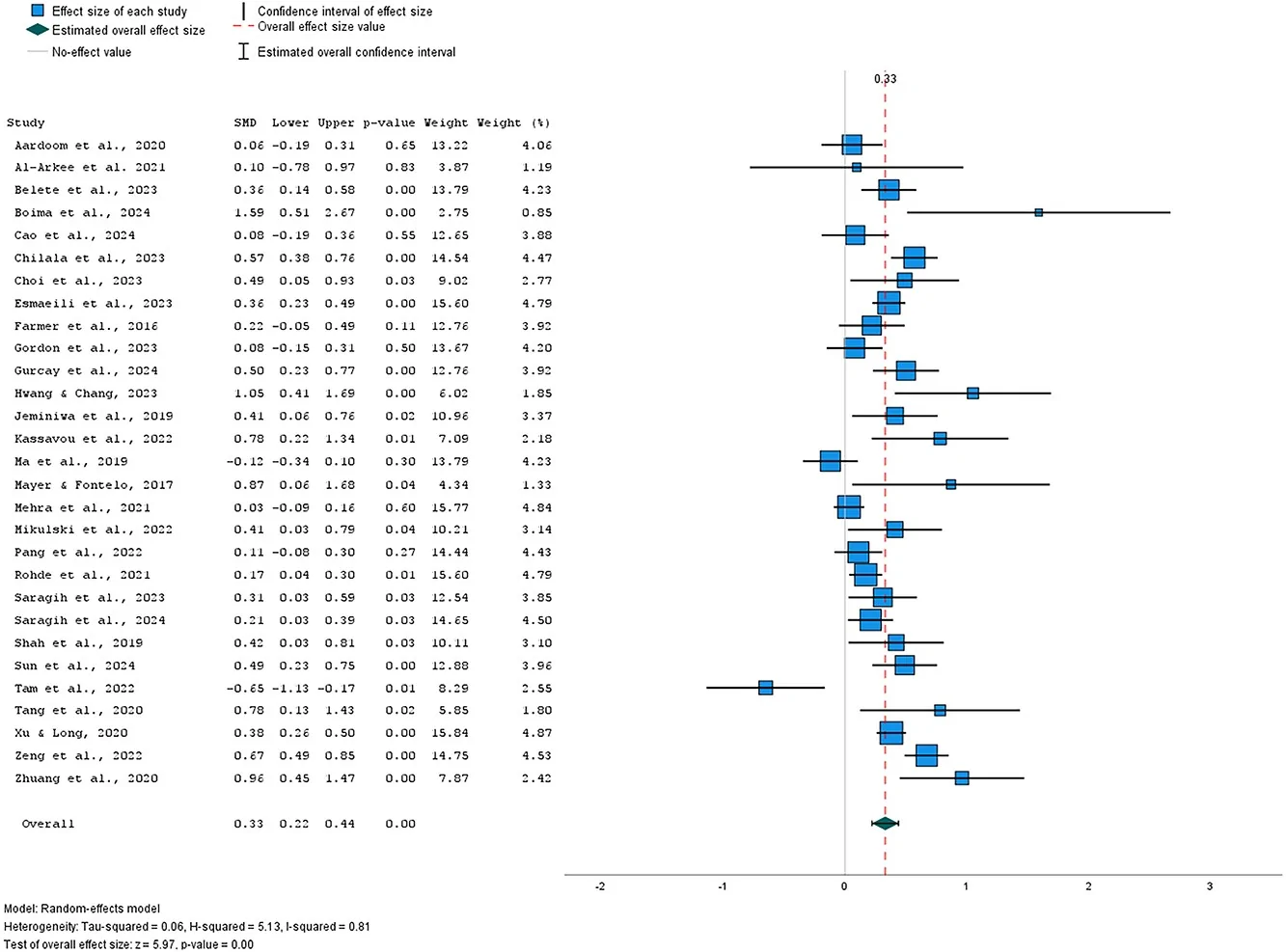
Forest plot of effect based on SMDs on medication adherence.

Meta-analyses based on SMDs showed a statistically significant small effect of patient-facing DHIs on medication adherence at post-intervention (SMD = 0.33, 95% CI = 0.22 to 0.44, *P* < .01, 29 meta-analyses, 71 438 participants). In the forest plot shown in Fig. 2, twenty studies reported a significant effect, one study (Tam et al. 2022) non-significant, and eight studies (Aardoom et al. 2020, Al-Arkee et al. 2021, Cao et al. 2024, Farmer et al. 2016, Gordon and Liu 2023, Ma et al. 2019, Mehra et al. 2021, Pang et al. 2022) were uncertain with high heterogeneity (I^2^ = 81%). The significantly effective interventions included those using mHealth using text messaging to improve medication adherence (Belete et al. 2023, Boima et al. 2024, Kassavou et al. 2022, Mayer and Fontelo 2017, Mikulski et al. 2022, Shah et al. 2019, Xu and Long 2020, Zeng et al. 2022, Zhuang et al. 2020), Telehealth or eHealth (Chilala et al. 2023, Esmaeili et al. 2023, Gurcay et al. 2024, Rohde et al. 2021, Saragih et al. 2023, Saragih et al. 2024, Sun et al. 2024, Tang et al. 2020), a combination of mHealth, Telehealth and/or eHealth interventions (Choi et al. 2023, Hwang and Chang 2023, Jeminiwa et al. 2019). There was no publication bias identified by Egger’s test with a *P*-value of .34.

**Figure 3:**
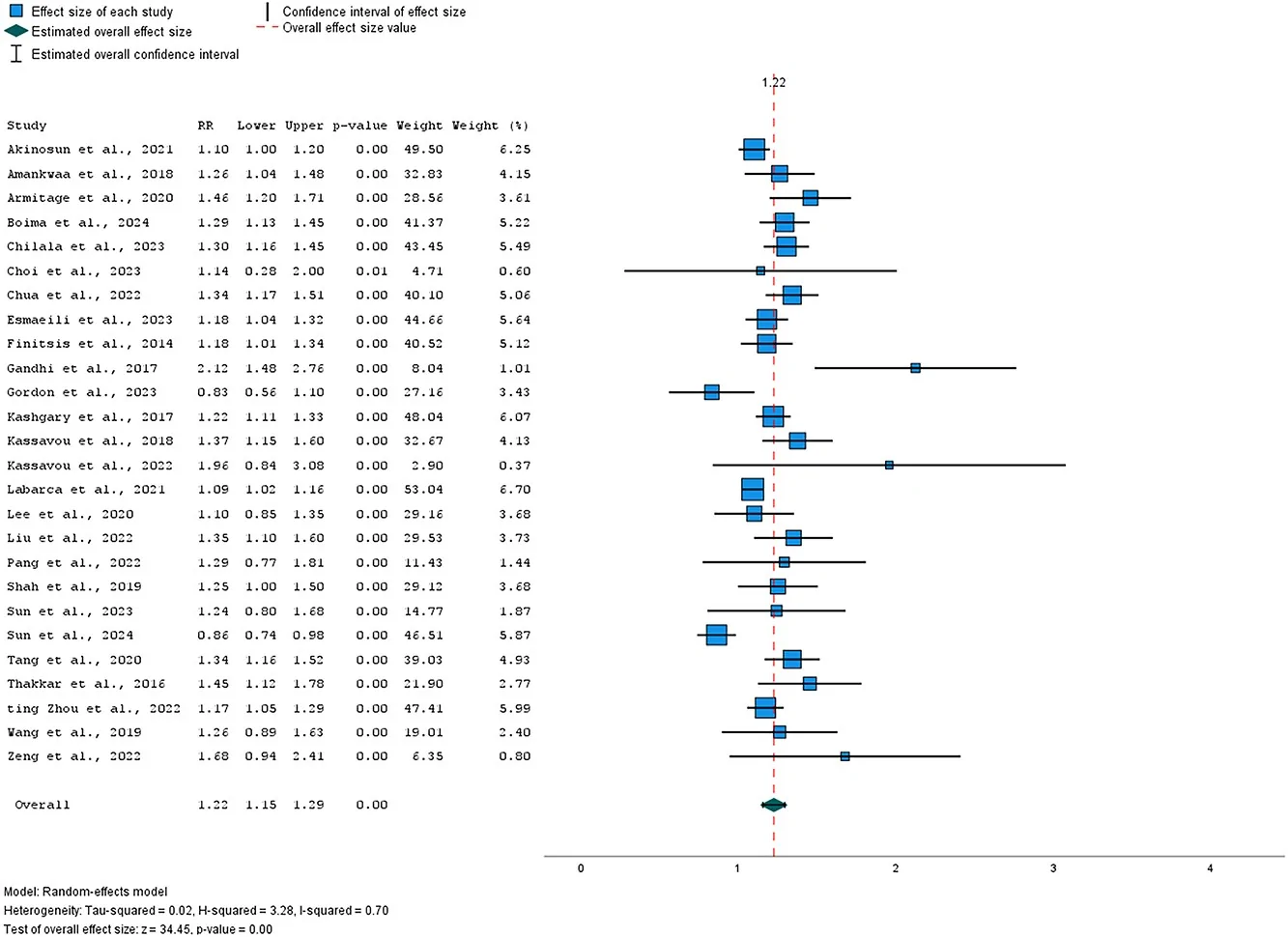
Forest plot of effect based on RRs on medication adherence.

In the pooled meta-analysis based on RRs, digital health interventions improved medication adherence (RR = 1.22; 95% CI = 1.15 to 1.29; *P* < .01; 26 meta-analyses, 90 264 participants). The forest plot in Fig. 3 illustrates that 24 studies reported a significant improvement in medication adherence among the treatment group following digital health interventions; however, two studies (Gordon and Liu 2023, Sun et al. 2024) indicated a significant reduction in medication adherence, accompanied by substantial heterogeneity (I^2^ = 70%). Publication bias was detected by Egger’s test with a *P*-value of <.001. The JBI quality appraisal regarding this outcome highlighted that the majority of the studies demonstrated strong or moderate quality (Table S1—Appendix I).

### Healthcare utilization

**Figure 4:**
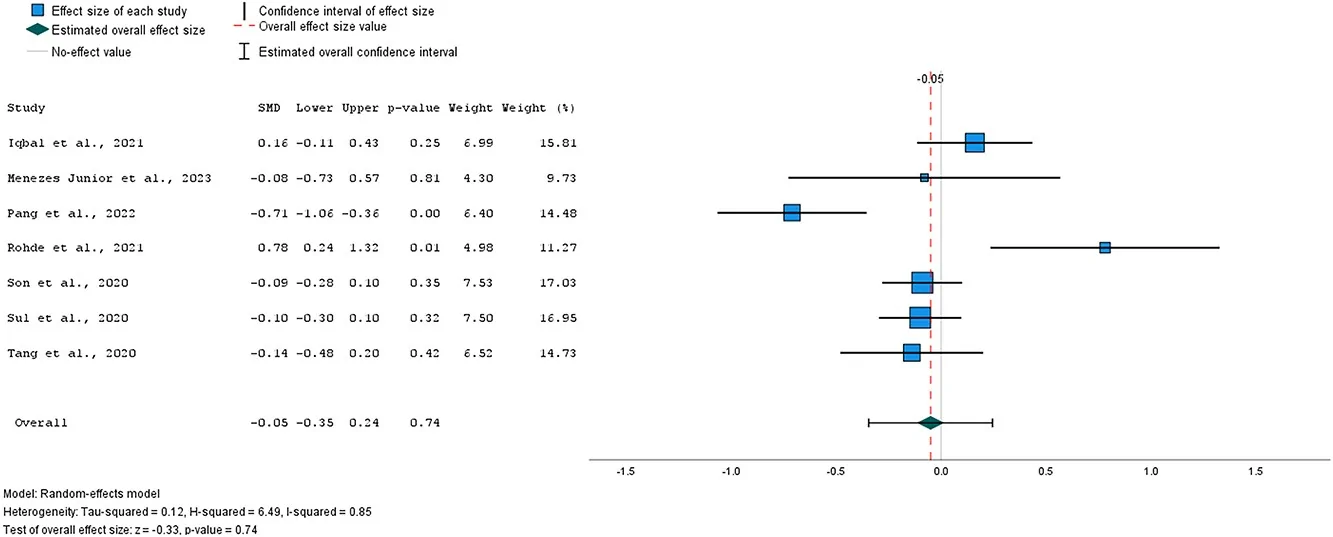
Forest plot of effect based on SMDs on healthcare utilization.

Meta-analyses based on SMDs indicated that patient-facing DHIs did not have a statistically significant effect on healthcare utilization post-intervention (SMD = −0.05, 95% CI = −0.35 to 0.24, *P* = .74, 7 meta-analyses, 12 396 participants). As shown in the forest plot in Fig. 4, one study (Rohde et al. 2021) reported a significant effect, one study (Pang et al. 2022) non-significant results, and five studies (Tang et al. 2020, Iqbal et al. 2021, Menezes Junior et al. 2023, Son et al. 2020, Sul et al. 2020) yielded uncertain results with high heterogeneity (I^2^ = 85%). The only statistically significant effective intervention was reported by Rohde et al. (2021), which showed that eHealth was effective in reducing the number of patient visits to clinics/hospitals.

**Figure 5:**
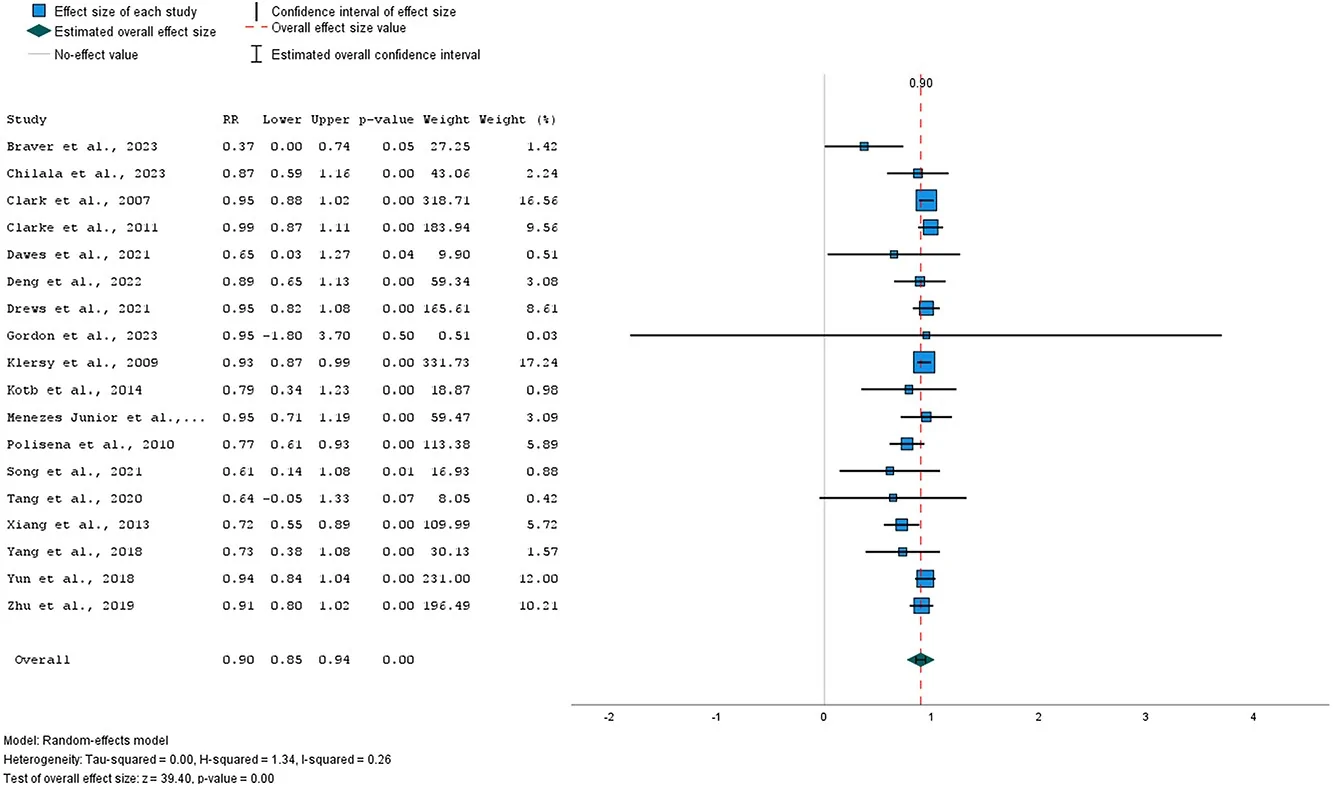
Forest plot of effect based on RRs on healthcare utilization.

In the pooled meta-analysis based on RRs, digital health interventions showed a lower risk for healthcare utilization than the usual care group (RR = 0.90; 95% CI = 0.85 to 0.94; *P* < .01; 18 meta-analyses, 39 907 participants). As shown in the forest plot in Fig. 5, 16 studies reported a significant effect on healthcare utilization among the treatment group following digital health interventions; however, two studies (Gordon and Liu 2023, Tang et al. 2020) were uncertain with moderate heterogeneity (I^2^ = 27%), although the majority of the studies presented strong or moderate quality according to the JBI quality appraisal tool (Table S1—Appendix I). Publication bias was detected by Egger’s test with a *P*-value of <.001.

### Falls

**Figure 6:**
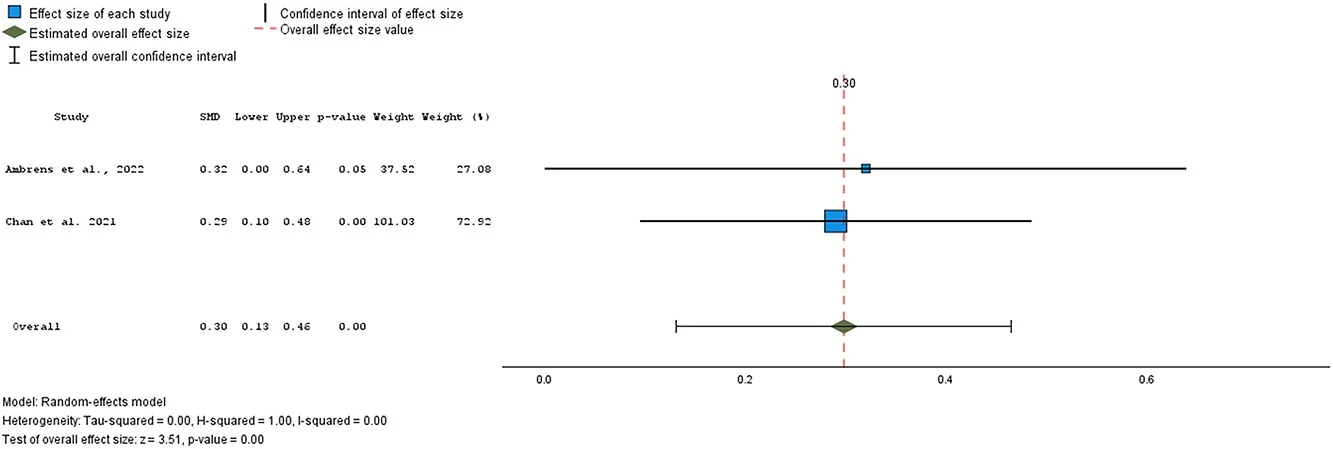
Forest plot of effect based on SMDs on falls.

Pooled analysis of two meta-analyses based on SMDs demonstrated a statistically significant small effect of patient-facing DHIs on reducing falls at post-intervention (SMD = 0.30, 95% CI = 0.13 to 0.46, *P* < .01, 1252 participants). As shown in the forest plot in Fig. 6, both studies (Ambrens et al. 2022, Chan et al. 2021) reported a significant effect with no heterogeneity (I^2^ = 0%). These studies evaluated the effectiveness of eHealth-delivered exercise programs (Ambrens et al. 2022) and e-interventions, including Telehealth and smart home systems, in reducing falls among older adults (Chan et al. 2021).

**Figure 7:**
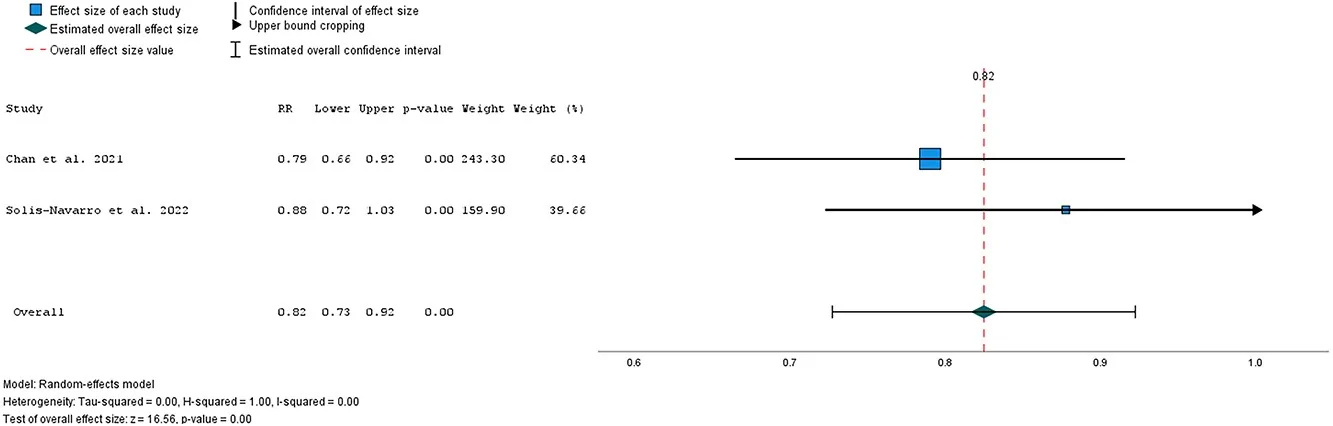
Forest plot of effect based on RRs on falls.

In the pooled meta-analysis based on RRs, digital health interventions indicated a lower risk of falls in the intervention group compared with the usual care group (RR = 0.82; 95% CI = 0.73 to 0.92; *P* < .01; 2 meta-analyses, 4631 participants). The forest plot in Fig. 7 illustrates that both included studies reported a significant reduction in falls among older adults following digital health interventions with no heterogeneity (I^2^ = 0%), alongside a strong/moderate quality according to the JBI quality appraisal tool (Table S1—Appendix I).

### Adverse events

**Figure 8:**
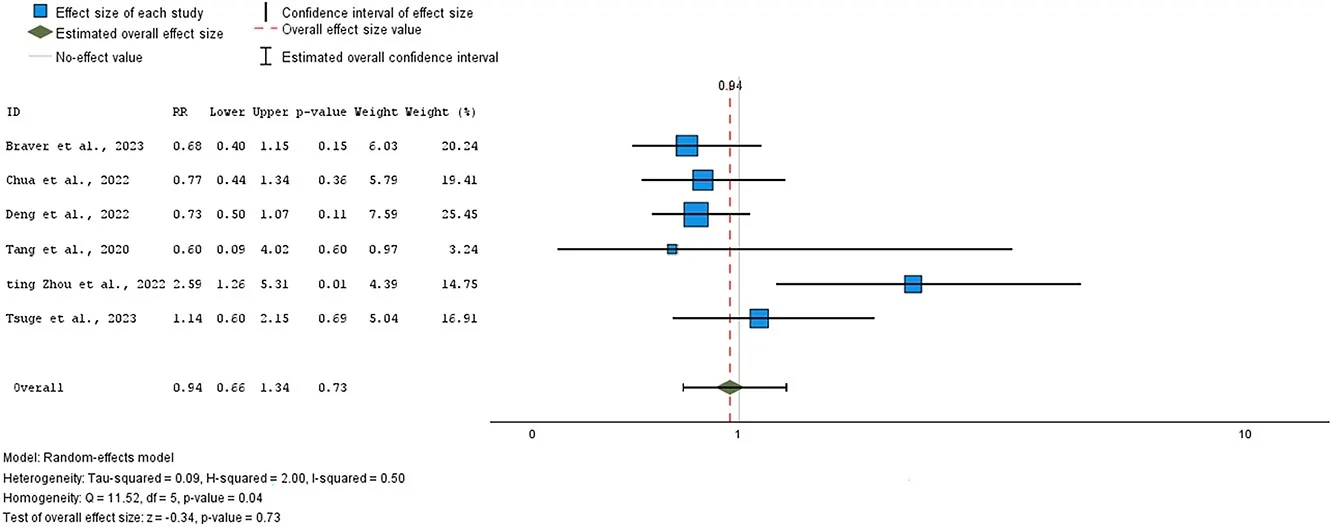
Forest plot of effect based on RRs on adverse events.

There were no meta-analysis studies evaluating the adverse events outcome as a continuous variable. However, six studies analysed adverse events as a dichotomous variable. Pooled analysis of RRs showed no statistically significant association between patient-facing DHIs and adverse events (RR = 0.94; 95% CI = 0.66 to 1.34; *P* = .73; 6 meta-analyses, 3460 participants). As shown in the forest plot in Fig. 8, only one study (Ting Zhou et al. 2022) reported a significant effect; the remaining five studies (Tang et al. 2020, Braver et al. 2023, Chua et al. 2022, Deng et al. 2022, Tsuge et al. 2023) were non-significant. Overall, the studies considered for this outcome showed moderate heterogeneity (I^2^ = 50%), and most of them strong/moderate quality according to the JBI quality appraisal tool (Table S1—Appendix I).

### Medication errors

No study analysed the medication error outcome as a continuous variable. Only one study (Ting Zhou et al. 2022) reported this outcome as a dichotomous variable; thus, there were not enough studies to conduct a meta-analysis for this dichotomous measurement.

### Mortality

**Figure 9:**
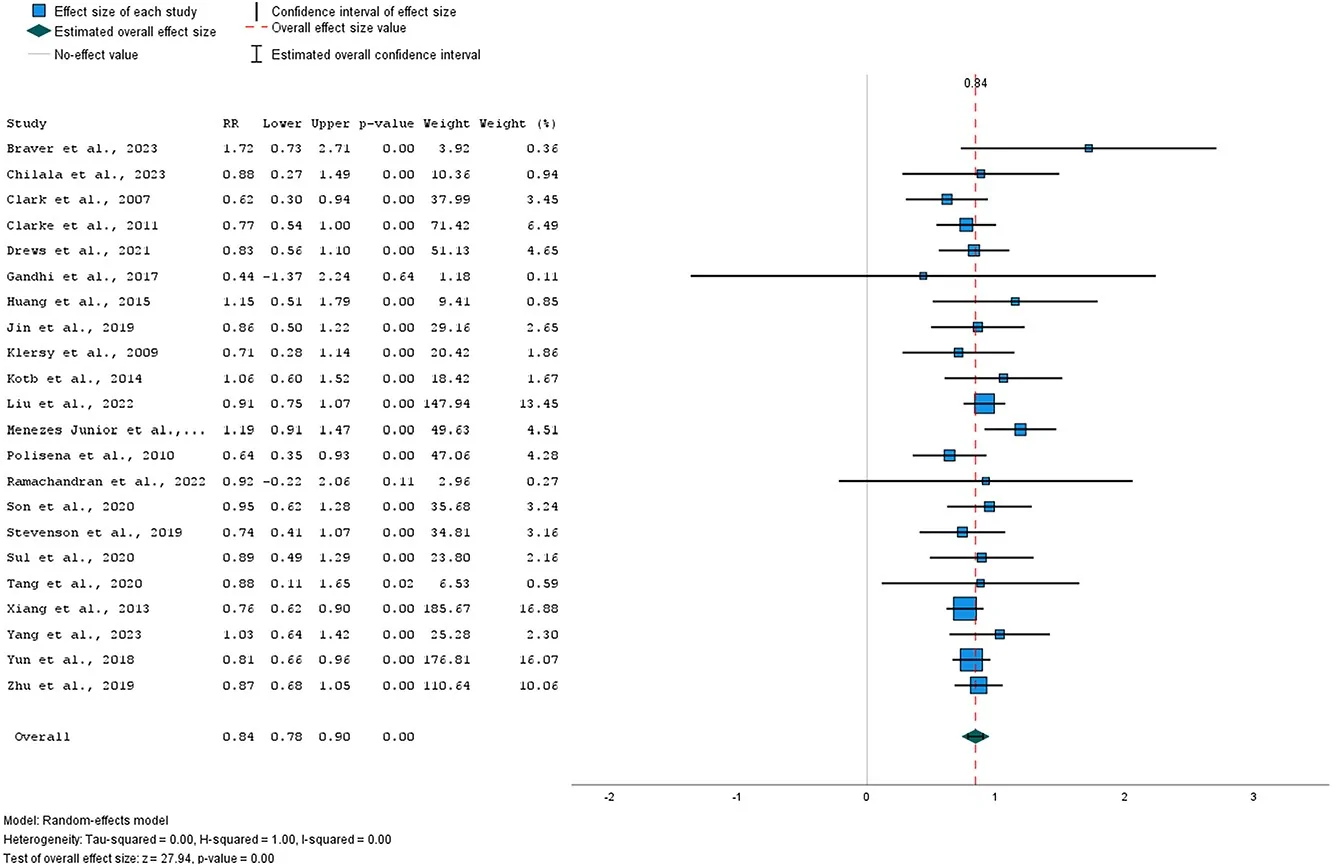
Forest plot of effect based on RRs on mortality.

Only one study (Iqbal et al. 2021) measured the mortality outcome as a continuous variable; thus, there were not enough studies to conduct a meta-analysis for this continuous measurement. However, 22 studies measured mortality as a dichotomous variable. Results of these meta-analyses based on RRs showed that the risk of mortality was lower in the intervention group than the usual care group (RR = 0.84; 95% CI = 0.78 to 0.90; *P* < .01; 22 meta-analyses, 64 968 participants). The forest plot in Fig. 9 shows that most studies (*n* = 15) reported a significant reduction in mortality following patient-facing DHIs. Five studies (Menezes Junior et al. 2023, Braver et al. 2023, Huang et al. 2015, Kotb et al. 2014, Yang et al. 2023) reported non-significant results. Two studies (Gandhi et al. 2017, Ramachandran et al. 2022) were uncertain, with no heterogeneity (I^2^ = 0%). The majority of the studies presented high/moderate quality according to the JBI quality appraisal tool (Table S1—Appendix I). However, a proportion of them also showed low or very low quality. A risk of publication bias was identified by Egger’s test with a *P*-value of <.001.

### Relapse rate

**Figure 10:**
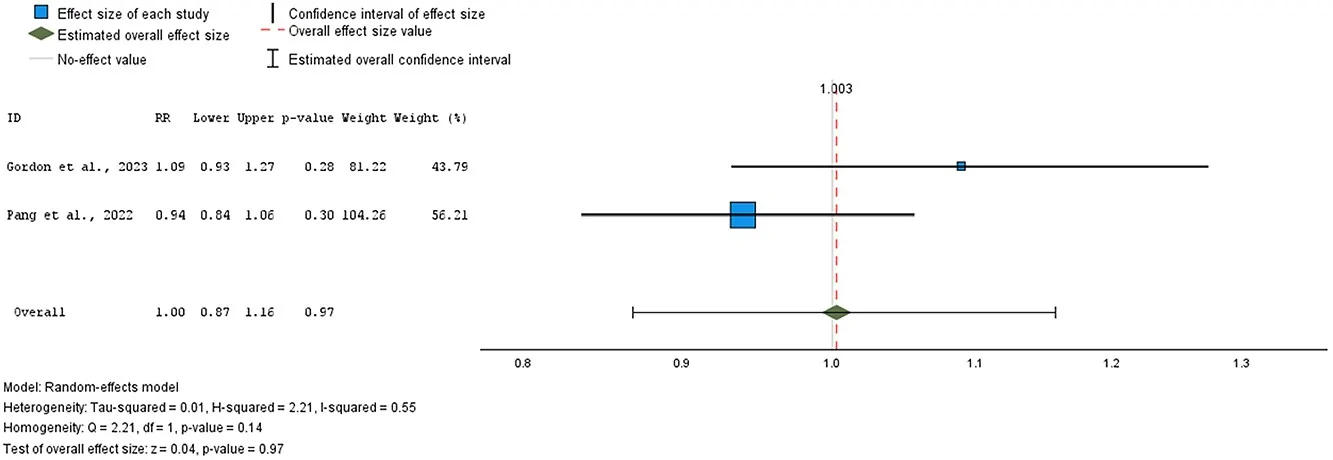
Forest plot of effect based on RRs on relapse rate.

Only one study (Huang et al. 2014) measured the relapse rate outcome as a continuous variable; thus, there were not enough studies to conduct a meta-analysis for this continuous measurement. However, two studies measured relapse rate as a dichotomous variable. In the pooled analysis based on RRs, patient-facing DHIs showed no association with relapse rate (RR = 1.00; 95% CI = 0.87 to 1.16; *P* = .97; 2 meta-analyses, 1642 participants). As shown in the forest plot in Fig. 10, neither study reached statistical significance individually [Gordon and Liu (2023): RR = 1.09, *P* = .28; Pang et al. (2022): RR = 0.94, *P* = .30], with substantial heterogeneity (I^2^ = 55%). Overall, the JBI quality appraisal showed strong quality of the studies regarding this outcome (Table S1—Appendix I).

### Readmissions

**Figure 11:**
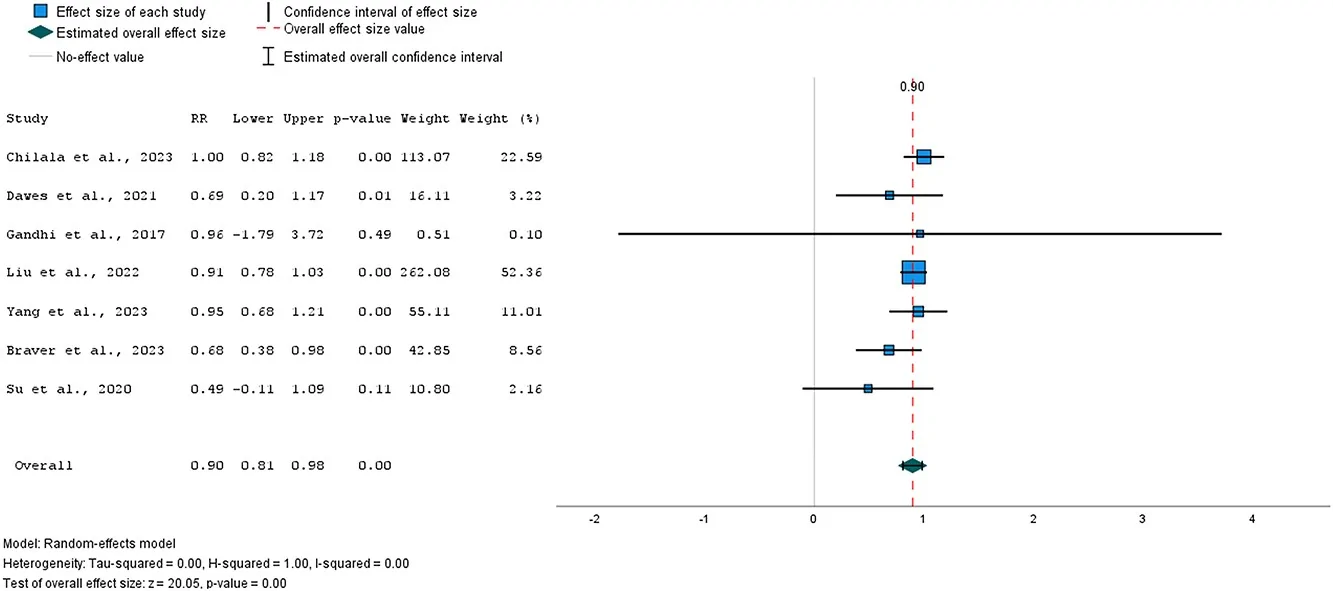
Forest plot of effect based on RRs on readmission.

Only one study (Son et al. 2020) measured the readmission outcome as a continuous variable; thus, there were not enough studies to conduct a meta-analysis for this continuous measurement. However, seven studies measured readmissions as a dichotomous variable. Results of these meta-analyses based on RRs showed that the risk of readmission was lower in the intervention group than the usual care group (RR = 0.90; 95% CI = 0.81 to 0.98; *P* < .01; 7 meta-analyses, 15 926 participants). The forest plot in Fig. 11 shows that four studies (Braver et al. 2023, Yang et al. 2023, Dawes et al. 2021, Liu et al. 2022) reported a significantly lower risk of readmission following patient-facing DHIs, one study (Rohde et al. 2021) a significantly higher risk and two studies (Gandhi et al. 2017, Su et al. 2020) were uncertain, with no heterogeneity (I^2^ = 0%). The JBI quality appraisal tool revealed that the majority of the studies presented strong quality (Table S1—Appendix I).

## Discussion

This study aimed to evaluate the effectiveness of patient-facing DHIs in improving patient safety outcomes through a meta-meta-analysis. As digital health technologies become increasingly integrated into healthcare systems and nursing care worldwide, it is crucial to assess their impact on key safety outcomes such as medication adherence, healthcare utilization, fall prevention, mortality reduction, and adverse events. The findings from this study demonstrate both the potential benefits and the current challenges of DHIs and highlight the most patient safety-sensitive outcomes affected by DHIs actively involving patients.

In detail, the findings suggest that patient-facing DHIs are effective in enhancing medication adherence, lowering fall risk in older adults, and reducing both mortality and hospital readmissions. On the contrary, patient-facing DHIs’ influence on healthcare utilization, medication errors and relapse rates appears inconsistent, with some studies indicating higher rates of patient visits, and mixed outcomes on medication errors. No significant association was found between patient-facing DHIs and adverse events or relapse rate, suggesting that, on the current evidence, these technologies do not measurably increase risk in either domain. In this vein, the controversial and complex aspects of the implementation of DHIs in healthcare and nursing care point clearly out the relevance of considering established frameworks for their adoption in order to maximize their effectiveness and safety and minimize their potential harms. Accordingly, healthcare professionals’ continuous educational development and organizational strategies to support the shift toward digital innovations in healthcare are key to achieving these benefits, alongside promoting patients’ empowerment and education to effectively adopt these solutions (Jarva et al. 2024, Kulju et al. 2024). Many patient safety outcomes affected by patient-facing DHIs are also nurse-sensitive outcomes. In detail, nursing care plays a crucial role in promoting medication adherence, preventing falls and medication errors, reducing mortality and readmissions, and providing appropriate healthcare utilization. Therefore, the integration of patient-facing DHIs into nursing care is a major contemporary challenge to ensure patient safety and effective care.

While the results of this meta-meta-analysis are well-supported by the high proportion of strong/moderate quality studies across all outcomes, it is important to highlight that several outcomes were affected by considerable heterogeneity. In detail, the meta-analytic synthesis regarding medication adherence demonstrated substantial heterogeneity, adverse events and relapse rate moderate-to-substantial heterogeneity, and healthcare utilization moderate heterogeneity. The remaining outcomes (falls, mortality and readmissions) were not affected by heterogeneity. Therefore, it is important to interpret with caution the results where heterogeneity was high or substantial. While a sub-group analysis might contribute in reducing heterogeneity, this option was methodologically excluded by design in order to enhance the comprehensiveness of this study (Biondi 2016). Moreover, several outcomes were underpinned by a small number of eligible studies. Hence, from the empirical point of view, dividing these already limited number of studies by intervention subtype or patient population would have left most subgroups with only one or two meta-analyses, producing estimates with wide CIs and a high risk of unstable findings. The comprehensive overview provided by grouping the studies by outcome is, therefore, the most robust and informative synthesis to inform research and practice on this topic, although heterogeneity should be considered when interpreting the results concerning some outcomes. This study also benefits from a low overlap between studies making its coverage of the current evidence more comprehensive. Overall, this study contributed in identifying the safety outcomes which are positively affected by patient-facing DHIs, and it has also identified inconclusive evidence, therefore providing useful insights for future research.

### Positive effects of patient-facing digital health interventions on safety outcomes

One of the most significant findings of this study is the positive impact of patient-facing DHIs on medication adherence. The results indicated a small but statistically significant improvement, suggesting that digital interventions such as mobile health (mHealth) applications, SMS reminders, and telehealth consultations contribute to increased adherence. This aligns with previous research demonstrating that digital reminders enhance adherence among patients with chronic illnesses (Boima et al. 2024, Kassavou and Sutton 2018). A similar meta-analysis found that mobile-based interventions significantly improved adherence, especially among patients undergoing chemotherapy (Akingbade et al. 2023). However, despite these positive effects, variations in effectiveness across different patient populations and healthcare settings suggest that user engagement and digital literacy play a critical role in determining outcomes (Causio et al., 2025). These findings highlight the necessity of tailoring digital adherence interventions to meet specific patient needs and integrating behavioral support strategies. While the majority of the studies demonstrated strong/moderate quality, these aspects should be interpreted by also considering the substantial heterogeneity found among these studies. In this vein, while the overall effectiveness of patient-facing DHIs on promoting medication adherence is supported, it is also recommended to explore specific DHIs in relation to specific health conditions and patient populations.

The results also demonstrated that patient-facing DHIs significantly reduced the risk of falls, particularly among older adults. Digital exercise programs, tele-rehabilitation, and smart home monitoring systems were particularly effective in preventing falls, corroborating the findings of Pereira et al. (2008), who concluded that eHealth interventions significantly decrease fall risks. However, real-world adoption of such interventions remains a challenge. Zhang (2023) found that older adults faced difficulties using digital fall prevention tools due to low digital literacy and limited accessibility. This suggests that while digital interventions can be effective, their real-world impact depends on ensuring user-friendliness and accessibility, particularly for aging populations. Future research should focus on developing more intuitive and inclusive digital health solutions tailored to older adults. The absence of heterogeneity regarding falls prevention, together with the strong/moderate quality of the studies, further support the effectiveness of these digital solutions on this outcome. As both medication adherence and falls prevention are considered as nurse-sensitive outcome (McEvoy et al. 2025), investing in educational programs to enhance nurses’ digital competences would be key to further consolidate these positive effects of patient-facing DHIs.

Furthermore, the study found a significant reduction in mortality rates among patients using patient-facing DHIs. This result is further supported by the absence of heterogeneity and the high proportion of strong/moderate quality studies, although some of them also demonstrated low/very low quality. From a wider perspective, this finding aligns with global research highlighting the role of predictive analytics, AI, and remote monitoring in improving survival rates in critical care settings. For instance, it has been demonstrated that AI-driven predictive modeling significantly enhances the early detection of patient deterioration, leading to improved survival outcomes (De Micco et al. 2025, Lester et al. 2025). Similarly, Schmidt et al. (2024) found that digital interventions in cardiac care led to significant mortality reductions due to enhanced monitoring and early intervention capabilities. However, disparities in implementation across healthcare systems may affect outcomes. Research by Marra et al. (2024) suggested that while digital interventions had the potential to reduce mortality, infrastructure limitations and interoperability challenges restricted their effectiveness. These findings emphasize the importance of integrating robust digital ecosystems and ensuring seamless interoperability between digital health platforms to maximize patient safety benefits.

Finally, patient-facing DHIs were also associated with reduced hospital readmission rates, aligning with research highlighting the role of telehealth and remote monitoring in post-discharge care (Dawes et al. 2021, Liu et al. 2022). Digital follow-up interventions have proven particularly effective in reducing readmission risks for chronic disease patients (Yang et al. 2018). While this meta-analytic result is further supported by the absence of heterogeneity and the quality of the studies, inconsistencies in previous evidence suggest that, while digital solutions improve post-discharge monitoring, factors such as patient adherence, healthcare provider engagement, and integration into routine care workflows influence their effectiveness (Gandhi et al. 2017). These results emphasize the need for comprehensive digital care models that extend beyond initial interventions to provide long-term support. Mortality and readmission rates are complex outcomes in healthcare, while the nursing care contributes to these outcomes (Needleman et al. 2020), implementing patient-facing DHIs and supporting digital health competences among healthcare professionals and nurses is key to further mitigate these outcomes and, hence, enhancing patient safety (Chauhan and McAlister 2022).

### Inconclusive effects of patient-facing digital health interventions on safety outcomes

The impact of patient-facing DHIs on healthcare utilization was found to be inconsistent and affected by moderate heterogeneity, with no significant reduction in hospital visits observed in the overall analysis. While telehealth and remote monitoring were expected to decrease unnecessary hospital visits, the findings suggest that accessibility to digital healthcare may, in some cases, lead to increased healthcare utilization. This finding is consistent with the work of Reno et al. (2022), who found that telehealth services reduced emergency department visits during the COVID-19 pandemic but did not sustain this trend in the post-pandemic period. Additionally, Darley et al., 2022 reported that increased digital engagement in the United Kingdom’s National Health Service led to higher outpatient visit rates, reflecting a paradox where improved access to care increases patient interactions rather than reducing utilization. These discrepancies underscore the complexity of digital health implementation and the need for refined strategies to balance accessibility with efficiency. The studies regarding this outcome showed strong/moderate quality suggesting a solid evidence base in supporting this finding.

Similarly, this study found limited evidence regarding the impact of patient-facing DHIs on medication errors, with only one study reporting this outcome. While DHIs, such as electronic prescribing and AI-driven decision support, have been shown to reduce medication errors in previous research (De Micco et al. 2025), the current meta-analysis highlights a gap in the literature due to the lack of sufficient data. Future research should explore in more depth the role of AI-driven clinical decision support systems in minimizing medication errors, ensuring that digital tools enhance accuracy rather than contribute to errors. Recent evidence has highlighted how AI-driven digital solutions might both create unnecessary double-checks in clinical decision making, as a safeguard measure against potential AI flaws (Lester et al. 2025), and promote safer healthcare environments through the early detection of adverse events (De Micco et al. 2025).

Also, the findings on relapse rates were inconclusive, with some studies indicating an increased risk of relapse in intervention groups while others reported significant reductions. Although the study quality was strong, this variability, further supported by substantial heterogeneity among these studies, aligns with prior research indicating that while DHIs improve disease management, adherence to self-management programs plays a crucial role in determining effectiveness (Gordon and Liu 2023, Pang et al. 2022). A study by Huang et al. (2014) similarly found that digital interventions reduced relapse rates among psychiatric patients but were less effective in other chronic disease populations. These results suggest that the success of patient-facing DHIs in preventing relapse depends on patient engagement, intervention design, and ongoing support mechanisms. In this vein, healthcare professionals and nurses’ digital health competences are again key in promoting proper access and utilization of DHIs among the population (Jarva et al. 2024). Furthermore, identifying healthcare professionals’ specific characteristics and profiles is key to designing the most effective strategies to promote the adoption of DHIs, alongside the organizational support provided by healthcare organizations and leaders. Recent research (Mikkonen et al. 2026) highlighted how digital competence is highly stratified, not only by competences but also by organizational culture, leadership, and peer influence. While younger and more educated healthcare professionals tend to demonstrate higher digital health competences, the major enabler of this pattern is a supportive environment, and leadership support is a major differentiator across competence profiles (Mikkonen et al. 2026).

Similarly, this study found no significant association between patient-facing DHIs and adverse events, in contrast to previous evidence regarding the safety risks of digital interventions (Jafleh et al., 2024). While one included study (Ting Zhou et al. 2022) reported a significant increase in adverse events, this was not consistent across the remaining five studies, and the pooled estimate showed no overall effect. This finding should still be interpreted in light of moderate heterogeneity and it aligns with the broader pattern identified across healthcare utilization, medication errors, and relapse rate, none of which showed a clear, consistent indication in either direction. Given this variability, continued rigorous evaluation of digital health tools remains warranted before widespread implementation, even in the absence of a statistically significant overall risk.

### Limitations and implications for future research

This study has several limitations. While the quantitative synthesis included a major proportion of strong quality studies and provides a solid overview of the main outcomes of patient-facing DHIs on patient safety, the lack of qualitative data synthesis may have resulted in missing additional insights, such as details regarding the type of interventions, the population, and other contextual factors that could provide a deeper understanding of the quantitative results. A qualitative synthesis is recommended for future research, and supplementary Appendix III (Table S2) reports the detailed list of the studies to be considered for this implementation, including references for both the systematic reviews and meta-analyses. The literature search was updated until June 2024. However, evidence on review currency suggests that most systematic reviews remain valid longer than 2 years with a median ‘survival’ time of 5.5 years before an update is needed (Shojania et al. 2007). Similarly, meta-analyses require substantial accumulation of primary studies before they can be conducted and published, so the pool of eligible meta-analyses evolves considerably more slowly than the primary literature itself. For this reason, while this study may benefit from future updates, currently, it still captures the most recent meta-analyses available on this topic. Additionally, while data on medication adherence, mortality, fall prevention, and readmissions were well-represented in the analysis, findings related to healthcare utilization, adverse events, medication errors, and relapse rates were inconclusive due to the limited number of studies or substantial heterogeneity. Regarding this aspect, we acknowledge that substantial heterogeneity observed in meta-analyses for medication adherence (substantial), and adverse events and relapse rate (moderate-to-substantial), reduces confidence in the precision of the pooled estimates. However, adopting a sub-group analysis would have affected the comprehensiveness of this synthesis and, the limited number of studies for some outcomes, would have also generated unstable and inaccurate measures affecting the overall solidity of the implications and evidence drawn from this study. The same applies to a sub-group analysis by quality level i.e. considering only studies with strong/moderate quality according to the JBI quality appraisal tool: while this would have strengthened the methodological rigor, it would also have reduced the comprehensiveness of this study and the stability of the results. At the same time, the heterogeneity found in this study reflects the same heterogeneity levels of previous meta-analyses performed on this research topic. In this vein, this study effectively captures the complexity and the variety of different patient-facing DHIs and related outcomes and their evolution in the literature over time. This meta-meta-analysis, for the first time, provides a comprehensive overview of this complexity by including a wide range of patient-facing DHIs and outcomes. In this regard, further research is needed to explore this complexity in more detail, especially the unintended consequences of DHIs. It is recommended that these outcomes be examined in future studies by considering specific digital solutions and health conditions, in order to further highlight the effectiveness of specific patient-facing DHIs on specific outcomes and groups of patients. Additionally, the study did not explore the role of digital literacy among patients and healthcare professionals. Barriers such as healthcare professionals’ education and digital health competences, patient engagement, and accessibility issues may influence real-world outcomes and should be considered. A health-economic perspective could also enhance this study, as it focuses on effectiveness rather than the cost-effectiveness of DHIs. Assessing whether these technologies offer a cost-effective solution for improving patient safety is an aspect that needs further investigation to inform policymakers and healthcare administrators.

## Conclusion

This study confirms the positive impact of patient-facing digital health interventions on patient safety outcomes. In detail, the results indicate that patient-facing DHIs significantly improve medication adherence, reduce the risk of falls among older adults, and decrease mortality and hospital readmission rates. However, the impact on healthcare utilization was inconsistent, with some studies reporting increased patient visits, while adverse events and relapse rate showed no significant association with patient-facing DHIs. These findings highlight concerns regarding the usability of patient-facing DHIs and suggest caution regarding the over-reliance on technology. In this vein, this study draws attention to some challenges and controversial effects of patient-facing DHIs with implications for their implementation in healthcare settings. Accordingly, it is key to consider the need for structured digital competency education among healthcare professionals and nurses to enhance the adoption and effective use of digital tools. This is also crucial to promote patients’ empowerment and the effective adoption of patient-facing DHIs by different categories of users.

Furthermore, research from low-resource settings highlights that infrastructure deficits continue to pose significant barriers to patients’ engagement and digital health implementation. Addressing these challenges requires a comprehensive approach that includes investment in education, user-centered design, and policy interventions to support widespread digital health adoption. Researchers and policy-makers should focus on optimizing digital interventions through tailored designs, improving healthcare professionals’ competences, and ensuring equitable access to digital health solutions across diverse populations. At the same time, it is key to include established frameworks in the implementation of patient-facing DHIs to ensure a safe and effective adoption of these solutions. Policy-makers should carefully consider the international bodies’ recommendations for addressing these challenges in order to maximize the potential of digital health interventions to enhance patient safety globally. Moreover, healthcare professionals’ digital health competence requires a supportive organizational culture, leadership, and peer influence. The integration of patient-facing DHIs in nursing care is imperative to provide patient safety and effective care.

## Supplementary Material

Supplementary_materials_oqag031

## Data Availability

Data supporting this study are available as supplementary files.

## References

[ref1] Aardoom JJ, Loheide-Niesmann L, Ossebaard HC et al. Effectiveness of eHealth interventions in improving treatment adherence for adults with obstructive sleep apnea: meta-analytic review. *J Med Internet Res* 2020;22:e16972. 10.2196/16972 PMC7055847 32130137

[ref2] Akingbade O, Nguyen KT, Chow KM. Effect of mHealth interventions on psychological issues experienced by women undergoing chemotherapy for breast cancer: a systematic review and meta-analysis. *J Clin Nurs* 2023;32:3058–73. 10.1111/jocn.16533 36168199

[ref3] Al-Arkee S, Mason J, Lane DA et al. Mobile apps to improve medication adherence in cardiovascular disease: systematic review and meta-analysis. *J Med Internet Res* 2021;23:e24190. 10.2196/24190 PMC8188316 34032583

[ref4] Ambrens M, Alley S, Oliveira JS et al. Effect of eHealth-delivered exercise programmes on balance in people aged 65 years and over living in the community: a systematic review and meta-analysis of randomised controlled trials. *BMJ Open* 2022;12:e051377. 10.1136/bmjopen-2021-051377 PMC9189851 35688586

[ref5] Aromataris E, Fernandez R, Godfrey CM et al. Summarizing systematic reviews: methodological development, conduct and reporting of an umbrella review approach. *Int J Evid-Based Healthc* 2015;13:132–40. 10.1097/XEB.0000000000000055 26360830

[ref6] Belete AM, Gemeda BN, Akalu TY et al. What is the effect of mobile phone text message reminders on medication adherence among adult type 2 diabetes mellitus patients: a systematic review and meta-analysis of randomized controlled trials. *BMC Endocr Disord* 2023;23:18. 10.1186/s12902-023-01268-8 PMC9850787 36658577

[ref7] Biondi-Zoccai G (ed). Umbrella Reviews: Evidence Synthesis with Overviews of Reviews and Meta-epidemiologic Studies. 1st edn. Cham, Switzerland. Springer International Publishing, 2016. 10.1007/978-3-319-25655-9

[ref8] Boima V, Doku A, Agyekum F et al. Effectiveness of digital health interventions on blood pressure control, lifestyle behaviours and adherence to medication in patients with hypertension in low-income and middle-income countries: a systematic review and meta-analysis of randomised controlled trials. EClinicalMedicine 2024;69. 10.1016/j.eclinm.2024.102432 PMC10850120 38333367

[ref9] Braver J, Marwick TH, Oldenburg B et al. Digital health programs to reduce readmissions in coronary artery disease: a systematic review and meta-analysis. *JACC: Adv* 2023;2:100591. 10.1016/j.jacadv.2023.100591 PMC11198697 38938339

[ref10] Cameron J, Ramaprasad A, Syn T. An ontology of mHealth. In: Proceedings of the 48th Hawaii International Conference on System Sciences, 2015, 1806–15.

[ref11] Cao W, Kadir AA, Tang W et al. Effectiveness of mobile application interventions for stroke survivors: systematic review and meta-analysis. *BMC Med Inform Decis Mak* 2024;24:6. 10.1186/s12911-023-02391-1 PMC10763083 38167316

[ref20] Causio FA, Gandolfi S, Kaur J, et al. (2025). Impact of digital health interventions on health literacy: A systematic review with quality appraisal. medRxiv. 10.1101/2025.02.27.25323025

[ref12] Chan JKY, Klainin-Yobas P, Chi Y et al. The effectiveness of e-interventions on fall, neuromuscular functions and quality of life in community-dwelling older adults: a systematic review and meta-analysis. *Int J Nurs Stud* 2021;113:103784. 10.1016/j.ijnurstu.2020.103784 33120138

[ref13] Chauhan U, McAlister FA. Comparison of mortality and hospital readmissions among patients receiving virtual ward transitional care vs usual post-discharge care: a systematic review and meta-analysis. *JAMA Netw Open* 2022;5:e2219113. 10.1001/jamanetworkopen.2022.19113 PMC9240908 35763296

[ref14] Chilala CI, Kassavou A, Sutton S. Evaluating the effectiveness of remote behavioral interventions facilitated by health care providers at improving medication adherence in cardiometabolic conditions: a systematic review and meta-analysis. *Ann Behav Med* 2023;57:99–110. 10.1093/abm/kaac037 35916782

[ref15] Choi YYC, Fineberg M, Kassavou A. Effectiveness of remote interventions to improve medication adherence in patients after stroke: a systematic literature review and meta-analysis. *Behav Sci* 2023;13:246. 10.3390/bs13030246 PMC10044982 36975271

[ref16] Chua BQY, Chong VWS, Teng TZJ et al. Does technology-enhanced communication improve helicobacter pylori eradication outcomes?—a meta-analysis. *Helicobacter* 2022;27:e12890. 10.1111/hel.12890 35363943

[ref16a] Clark RA, Inglis SC, McAlister FA et al. Telemonitoring or structured telephone support programmes for patients with chronic heart failure: systematic review and metaanalysis. Bmj 2007;334:942. 10.1136/bmj.39156.536968.55 PMC1865411 17426062

[ref17] Cleophas TJ, Zwinderman AH, Cleophas TJ et al. Meta-meta-analysis: a shift from a single to multiple meta-analyses. In: Cleophas JT, Zwinderman HA (eds.), Modern Meta-analysis: Review and Update of Methodologies. Cham: Springer International Publishing, 2017, 135–43. 10.1007/978-3-319-55895-0_11

[ref18] Cohen J . Statistical power analysis for the behavioral sciences. 2nd edn. Lawrence Erlbaum Associates, 1988.

[ref19] Covidence. Covidence systematic review software. Melbourne, Australia: Veritas Health Innovation, 2023.

[ref21] Darley S, Coulson T, Peek N, et al. Understanding how the design and implementation of online consultations affect primary care quality: systematic review of evidence with recommendations for designers, providers, and researchers. J Med Internet Res 2022;24:e37436. 10.2196/37436 PMC9621309 36279172

[ref22] Dawes AJ, Lin AY, Varghese C et al. Mobile health technology for remote home monitoring after surgery: a meta-analysis. *Br J Surg* 2021;108:1304–14. 10.1093/bjs/znab323 34661649

[ref23] De Micco F, Di Palma G, Ferorelli D et al. Artificial intelligence in healthcare: transforming patient safety with intelligent systems—a systematic review. *Front Med* 2025;11:1522554. 10.3389/fmed.2024.1522554 PMC11750995 39845830

[ref24] Deeks JJ, Higgins JPT, Altman DG. Analysing data and undertaking meta-analyses. In: Higgins JPT et al. (eds.), Cochrane handbook for systematic reviews of interventions. Cochrane, 2019, 241–84. 10.1002/9781119536604.ch10

[ref25] Deng LY, Wu Q, Ding F et al. The effect of telemedicine on secondary prevention of atherosclerotic cardiovascular disease: a systematic review and meta-analysis. *Front Cardiovasc Med* 2022;9:1020744. 10.3389/fcvm.2022.1020744 PMC9683074 36440018

[ref26] Donabedian A . The quality of care: how can it be assessed? *JAMA* 1988;260:1743–8. 10.1001/jama.260.12.1743 3045356

[ref27] Egger M, Smith GD, Schneider M et al. Bias in meta-analysis detected by a simple, graphical test. *BMJ* 1997;315:629–34. 10.1136/bmj.315.7109.629 PMC2127453 9310563

[ref28] Esmaeili ED, Azizi H, Dastgiri S et al. Does telehealth affect the adherence to ART among patients with HIV? A systematic review and meta-analysis. *BMC Infect Dis* 2023;23:169. 10.1186/s12879-023-08119-w PMC10022569 36932376

[ref29] Farmer AJ, McSharry J, Rowbotham S et al. Effects of interventions promoting monitoring of medication use and brief messaging on medication adherence for people with type 2 diabetes: a systematic review of randomized trials. *Diabet Med* 2016;33:565–79. 10.1111/dme.12987 26470750

[ref30] Gandhi S, Chen S, Hong L et al. Effect of mobile health interventions on the secondary prevention of cardiovascular disease: systematic review and meta-analysis. *Can J Cardiol* 2017;33:219–31. 10.1016/j.cjca.2016.08.017 27956043

[ref31] Gordon J, Sinopoulou V, Lakunina S et al. Remote care through telehealth for people with inflammatory bowel disease. Cochrane Database of Systematic Reviews 2023:CD014821. 10.1002/14651858.CD014821.pub2 PMC10164701 37140025

[ref32] Grygorian A, Montano D, Shojaa M et al. Digital health interventions and patient safety in abdominal surgery: a systematic review and meta-analysis. *JAMA Netw Open* 2024;7:e248555. 10.1001/jamanetworkopen.2024.8555 PMC11053376 38669018

[ref33] Gurcay B, Yilmaz FT, Bilgin A. The effectiveness of telehealth interventions on medication adherence among patients with type 2 diabetes: a meta-analysis. *Telemed E-Health* 2024;30:3–20. 10.1089/tmj.2023.0088 37219578

[ref34] Hackenbroich S, Kranke P, Meybohm P et al. Include or not to include conference abstracts in systematic reviews? Lessons learned from a large Cochrane network meta-analysis including 585 trials. *Syst Rev* 2022;11:178. 10.1186/s13643-022-02048-6 PMC9413929 36028879

[ref35] Haddaway NR, Collins AM, Coughlin D et al. The role of Google scholar in evidence reviews and its applicability to grey literature searching. *PLoS One* 2015;10:e0138237. 10.1371/journal.pone.0138237 PMC4574933 26379270

[ref36] Huang VW, Reich KM, Fedorak RN. Distance management of inflammatory bowel disease: systematic review and meta-analysis. *World J Gastroenterol* 2014;20:829–42. 10.3748/wjg.v20.i3.829 PMC3921492 24574756

[ref37] Huang K, Liu W, He D et al. Telehealth interventions versus center-based cardiac rehabilitation of coronary artery disease: a systematic review and meta-analysis. *Eur J Prev Cardiol* 2015;22:959–71. 10.1177/2047487314561168 25488550

[ref38] Hwang M, Chang AK. The effect of nurse-led digital health interventions on blood pressure control for people with hypertension: a systematic review and meta-analysis. *J Nurs Scholarsh* 2023;55:1020–35. 10.1111/jnu.12882 36929538

[ref39] IBM Corp . IBM SPSS Statistics for Windows (Version 28.0). IBM Corp, 2021

[ref40] Iqbal FM, Lam K, Joshi M et al. Clinical outcomes of digital sensor alerting systems in remote monitoring: a systematic review and meta-analysis. *NPJ Digit Med* 2021;4:7. 10.1038/s41746-020-00378-0 PMC7794456 33420338

[ref73] Jafleh EA, Alnaqbi FA, Almaeeni HA et al. The role of wearable devices in chronic disease monitoring and patient care: a comprehensive review. Cureus 2024;16:e68921. 10.7759/cureus.68921 PMC11461032 39381470

[ref41] Jarva E, Oikarinen A, Andersson J et al. Healthcare professionals’ digital health competence profiles and associated factors: a cross-sectional study. *J Adv Nurs* 2024;80:3236–52. 10.1111/jan.16096 38323687

[ref42] Jeminiwa R, Hohmann L, Qian J et al. Impact of eHealth on medication adherence among patients with asthma: a systematic review and meta-analysis. *Respir Med* 2019;149:59–68. 10.1016/j.rmed.2019.02.011 30803887

[ref43] Joanna Briggs Institute . Checklist for systematic reviews and research syntheses. JBI, 2017. https://jbi.global/sites/default/files/2019-05/JBI_Critical_Appraisal-Checklist_for_Systematic_Reviews2017_0.pdf

[ref44] Johnston BC, Patrick DL, Thorlund K et al. Patient-reported outcomes in meta-analyses—part 2: methods for improving interpretability for decision-makers. *Health Qual Life Outcomes* 2013;11:211. 10.1186/1477-7525-11-211 PMC3984637 24359184

[ref45] Kassavou A, Sutton S. Automated telecommunication interventions to promote adherence to cardio-metabolic medications: meta-analysis of effectiveness and meta-regression of behaviour change techniques. *Health Psychol Rev* 2018;12:25–42. 10.1080/17437199.2017.1365617 28805162

[ref46] Kassavou A, Wang M, Mirzaei V et al. The association between smartphone app–based self-monitoring of hypertension-related behaviors and reductions in high blood pressure: systematic review and meta-analysis. *JMIR mHealth uHealth* 2022;10:e34767. 10.2196/34767 PMC9328789 35819830

[ref16b] Klersy C, De Silvestri A, Gabutti G et al. A meta-analysis of remote monitoring of heart failure patients. J Am Coll Cardiol 2009;54:1683–94. 10.1016/j.jacc.2009.08.017 19850208

[ref47] Konttila J, Siira H, Kyngäs H et al. Healthcare professionals’ competence in digitalisation: a systematic review. *J Clin Nurs* 2019;28:745–61. 10.1111/jocn.14710 30376199

[ref48] Kotb A, Hsieh S, Wells GA. The effect of telephone support interventions on coronary artery disease (CAD) patient outcomes during cardiac rehabilitation: a systematic review and meta-analysis. *PLoS One* 2014;9:e96581. 10.1371/journal.pone.0096581 PMC4010507 24798429

[ref50] Kulju E, Jarva E, Oikarinen A et al. Educational interventions and their effects on healthcare professionals’ digital competence development: a systematic review. *Int J Med Inform* 2024;185:105396. 10.1016/j.ijmedinf.2024.105396 38503251

[ref51] Lester C, Rowell B, Zheng Y et al. Effect of uncertainty-aware AI models on pharmacists’ reaction time and decision-making in a web-based mock medication verification task: randomized controlled trial. *JMIR Med Inform* 2025;13:e64902. 10.2196/64902 PMC12023801 40249341

[ref52] Li X, Chen W. Enhancing digital competence: educational interventions in healthcare. *Comput Hum Behav* 2024;144:107734.

[ref53] Lima Júnior AJD, Zanetti ACB, Dias BM et al. Occurrence and preventability of adverse events in hospitals: a retrospective study. *Rev Bras Enferm* 2023;76:e20220025. 10.1590/0034-7167-2022-0025 PMC10332367 37436233

[ref54] Liu S, Li J, Wan DY et al. Effectiveness of eHealth self-management interventions in patients with heart failure: systematic review and meta-analysis. *J Med Internet Res* 2022;24:e38697. 10.2196/38697 PMC9555330 36155484

[ref55] Longhini J, Rossettini G, Palese A. Digital health competencies and affecting factors among healthcare professionals: additional findings from a systematic review. *J Res Nurs* 2024;29:156–76. 10.1177/17449871241226899 PMC11271674 39070573

[ref56] Ma Y, Cheng HY, Cheng L et al. The effectiveness of electronic health interventions on blood pressure control, self-care behavioural outcomes and psychosocial well-being in patients with hypertension: a systematic review and meta-analysis. *Int J Nurs Stud* 2019;92:27–46. 10.1016/j.ijnurstu.2018.11.007 30690164

[ref57] Marra C, Chico T, Alexandrow A et al. Addressing the challenges of integrating digital health technologies to measure patient-centred outcomes in clinical registries. *Lancet Digit Health* 2024;6:e50–9. 10.1016/S2589-7500(24)00223-1 39672737

[ref58] Mayer JE, Fontelo P. Meta-analysis on the effect of text message reminders for HIV-related compliance. *AIDS Care* 2017;29:409–17. 10.1080/09540121.2016.1214674 PMC5480218 27477580

[ref59] McEvoy NL, Kalvas LB, Walsh K et al. The identification and characterization of nurse-sensitive outcomes in acute and critical care: a systematic review. *Nurs Outlook* 2025;73:102379. 10.1016/j.outlook.2025.102379 39999613

[ref60] McGowan J, Sampson M, Salzwedel DM et al. PRESS peer review of electronic search strategies: 2015 guideline statement. *J Clin Epidemiol* 2016;75:40–6. 10.1016/j.jclinepi.2016.01.021 27005575

[ref61] Mehra N, Tunje A, Hallström IK et al. Effectiveness of mobile phone text message reminder interventions to improve adherence to antiretroviral therapy among adolescents living with HIV: a systematic review and meta-analysis. *PLoS One* 2021;16:e0254890. 10.1371/journal.pone.0254890 PMC8297901 34293033

[ref62] Menezes Junior AS, Rivera A, Ayumi Miyawaki I et al. Long-term remote vs. conventional monitoring of pacemakers: systematic review and meta-analysis of randomized controlled trials. *Curr Cardiol Rep* 2023;25:1415–24. 10.1007/s11886-023-01963-x 37751037

[ref63] Mikkonen K, Kaakinen P, Lahtinen M et al. Healthcare professionals’ competence in digitalisation: a systematic review. *J Clin Nurs* 2018;28:745–61. 10.1111/jocn.14710 30376199

[ref64] Mikkonen K, Tomietto M, Lee JJ et al. Digital health competence among healthcare professionals: a cross-sectional cluster analysis across 19 countries and regions. *Int J Nurs Stud* 2026;176:105348. 10.1016/j.ijnurstu.2026.105348 41621343

[ref65] Mikulski BS, Bellei EA, Biduski D et al. Mobile health applications and medication adherence of patients with hypertension: a systematic review and meta-analysis. *Am J Prev Med* 2022;62:626–34. 10.1016/j.amepre.2021.11.003 34963562

[ref66] Murray E, Hekler EB, Andersson G et al. Evaluating digital health interventions: key questions and approaches. *Am J Prev Med* 2016;51:843–51. 10.1016/j.amepre.2016.06.008 PMC5324832 27745684

[ref67] Needleman J, Liu J, Shang J et al. Association of registered nurse and nursing support staffing with inpatient hospital mortality. *BMJ Qual Saf* 2020;29:10–8. 10.1136/bmjqs-2018-009219 31391314

[ref68] Page MJ, McKenzie JE, Bossuyt PM et al. The PRISMA 2020 statement: an updated guideline for reporting systematic reviews. *BMJ* 2021;372:n71. 10.1136/bmj.n71 PMC8005924 33782057

[ref69] Pang LL, Liu HY, Liu ZD et al. Role of telemedicine in inflammatory bowel disease: systematic review and meta-analysis of randomized controlled trials. *J Med Internet Res* 2022;24:e28978. 10.2196/28978 PMC8990345 35323120

[ref70] Pereira CLN, Vogelaere P, Baptista F. Role of physical activity in the prevention of falls and their consequences in the elderly. *Eur Rev Aging Phys Act* 2008;5:51–8.

[ref71] Pieper D, Antoine SL, Mathes T et al. Systematic review finds overlapping reviews were not mentioned in every other overview. *J Clin Epidemiol* 2014;67:368–75. 10.1016/j.jclinepi.2013.11.007 24581293

[ref72] Pollock M, Fernandez R, Becker LA et al. Chapter V: overviews of reviews. Cochrane handbook for systematic reviews of interventions. *Cochrane* 2022;6.

[ref16c] Pollock M, Fernandes RM, Becker LA et al. 2020. Chapter V: Overviews of reviews. In Higgins JPT, Thomas J, Chandler J, et al. (eds.), Cochrane handbook for systematic reviews of interventions (version 6.0, updated March 2020). Cochrane.

[ref74] Ramachandran HJ, Jiang Y, San Tam WW et al. Effectiveness of home-based cardiac telerehabilitation as an alternative to phase 2 cardiac rehabilitation of coronary heart disease: a systematic review and meta-analysis. *Eur J Prev Cardiol* 2022;29:1017–43. 10.1093/eurjpc/zwab106 PMC8344786 34254118

[ref75a] Reno EM, Li BH, Eutermoser M et al. Temporal associations between emergency department and telehealth volumes during the COVID-19 pandemic: A time-series analysis from 2 academic medical centers. *Am J Emerg Med* 2022;54:238–41. 10.1016/j.ajem.2022.01.046 PMC8817426 35182918

[ref75] Richardson WS, Wilson MC, Nishikawa J et al. The well-built clinical question: a key to evidence-based decisions. *ACP J Club* 1995;123:A12–3. 10.7326/ACPJC-1995-123-3-A12 7582737

[ref76] Rohde JA, Barker JO, Noar SM. Impact of eHealth technologies on patient outcomes: a meta-analysis of chronic gastrointestinal illness interventions. *Transl Behav Med* 2021;11:1–10. 10.1093/tbm/ibz166 31731292

[ref77] Saragih ID, Schorr E, Porta CM et al. Effects of telehealth-assisted interventions for secondary prevention of cardiovascular disease: a systematic review and meta-analysis. *J Clin Nurs* 2023;32:3613–29. 10.1111/jocn.16452 35821631

[ref78] Saragih ID, Tonapa SI, Osingada CP et al. Effects of telehealth-assisted interventions among people living with HIV/AIDS: a systematic review and meta-analysis of randomized controlled studies. *J Telemed Telecare* 2024;30:438–50. 10.1177/1357633X211070726 34967240

[ref79] Schmidt KES, Waclawovsky G, dos Santos AM et al. Effect of a nonpharmacological psychological stress management intervention on major cardiovascular events and mortality in patients with coronary artery disease: a systematic review and meta-analysis of randomized clinical trials. *Int J Stress Manag* 2024;31:219–37. 10.1037/str0000327

[ref80] Shah R, Watson J, Free C. A systematic review and meta-analysis in the effectiveness of mobile phone interventions used to improve adherence to antiretroviral therapy in HIV infection. *BMC Public Health* 2019;19:915. 10.1186/s12889-019-6899-6 PMC6617638 31288772

[ref81] Shojania KG, Sampson M, Ansari MT et al. How quickly do systematic reviews go out of date? A survival analysis. *Ann Intern Med* 2007;147:224–33. 10.7326/0003-4819-147-4-200708210-00179 17638714

[ref82] Singh B, Ahmed M, Staiano AE et al. A systematic umbrella review and meta-meta-analysis of eHealth and mHealth interventions for improving lifestyle behaviours. *NPJ Digit Med* 2024;7:179. 10.1038/s41746-024-01172-y PMC11226451 38969775

[ref83] Slawomirski L, Klazinga N. The economics of patient safety: From analysis to action. OECD Health Policy Studies, 2022.

[ref84] Son Y-J, Lee Y, Lee H-J. Effectiveness of mobile phone-based interventions for improving health outcomes in patients with chronic heart failure: a systematic review and meta-analysis. *Int J Environ Res Public Health* 2020;17:1749. 10.3390/ijerph17051749 PMC7084843 32156074

[ref85] Su JJ, Yu DSF, Paguio JT. Effect of eHealth cardiac rehabilitation on health outcomes of coronary heart disease patients: a systematic review and meta-analysis. *J Adv Nurs* 2020;76:754–72. 10.1111/jan.14272 31769527

[ref86] Sul A-R, Lyu D-H, Park D-A. Effectiveness of telemonitoring versus usual care for chronic obstructive pulmonary disease: a systematic review and meta-analysis. *J Telemed Telecare* 2020;26:189–99. 10.1177/1357633X18811757 30541375

[ref87] Sun S, Zhang B, Zhang N et al. Effect of telehealth interventions on adherence to endocrine therapy among patients with breast cancer: a systematic review and meta-analysis. *Support Care Cancer* 2024;32:151. 10.1007/s00520-024-08339-z 38332357

[ref88] Tam HL, Leung LYL, Wong EML et al. Integration of text messaging interventions into hypertension management among older adults: a systematic review and meta-analysis. *Worldviews Evid-Based Nurs* 2022;19:16–27. 10.1111/wvn.12549 35014147

[ref89] Tang J, James L, Howell M et al. eHealth interventions for solid organ transplant recipients: a systematic review and meta-analysis of randomized controlled trials. *Transplantation* 2020;104:e224–35. 10.1097/TP.0000000000003294 32732828

[ref90] Ting Zhou T, Wang R, Jia Gu S et al. Effectiveness of mobile medical apps in ensuring medication safety among patients with chronic diseases: systematic review and meta-analysis. *JMIR mHealth uHealth* 2022;10:e39819. 10.2196/39819 PMC9727690 36413386

[ref91] Tsuge T, Yamamoto N, Taito S et al. Efficacy of telerehabilitation for patients after hip fracture surgery: a systematic review and meta-analysis. *J Telemed Telecare* 2023;31:174–83. 10.1177/1357633X231181632 37416946

[ref92] VanderWeele TJ . On a square-root transformation of the odds ratio for a common outcome. *Epidemiology* 2017;28:e58–60. 10.1097/EDE.0000000000000733 PMC5617805 28816709

[ref93] Veroniki A, Jackson D, Viechtbauer W et al. Methods to calculate uncertainty in the estimated overall effect size from a random-effects meta-analysis. *Res Synth Methods* 2019;10:23–43. 10.1002/jrsm.1319 30129707

[ref94] Vitija A, Amirthalingam A, Soltani A. The impact of digital interventions on medication adherence in paediatric populations with attention deficit hyperactivity disorder, depression, and/or anxiety: a rapid systematic review and meta-analysis. *Res Soc Adm Pharm* 2022;18:4017–27. 10.1016/j.sapharm.2022.07.042 35985977

[ref95] World Health Organization . Classification of Digital Health Interventions v1. 0: A Shared Language to Describe the Uses of Digital Technology for Health (No. WHO/RHR/18.06). Geneva: World Health Organization, 2018.

[ref96] World Health Organization . The Ongoing Journey to Commitment and Transformation: Global Patient Safety Action Plan 2021–2030. Geneva: World Health Organization, 2023.

[ref97] Xu H, Long H. The effect of smartphone app-based interventions for patients with hypertension: systematic review and meta-analysis. *JMIR mHealth uHealth* 2020;8:e21759. 10.2196/21759 PMC7605981 33074161

[ref98] Yang F, Wang Y, Yang C et al. Mobile health applications in self-management of patients with chronic obstructive pulmonary disease: a systematic review and meta-analysis of their efficacy. *BMC Pulm Med* 2018;18:147. 10.1186/s12890-018-0671-z PMC6122553 30180835

[ref99] Yang Z, Jia X, Li J et al. Efficacy and safety of hybrid comprehensive telerehabilitation (HCTR) for cardiac rehabilitation in patients with cardiovascular disease: a systematic review and meta-analysis of randomized controlled trials. *Occup Ther Int* 2023;2023:5147805. 10.1155/2023/5147805 PMC10432031 37593110

[ref100] Ye J, Woods D, Jordan N et al. The role of artificial intelligence for the application of integrating electronic health records and patient-generated data in clinical decision support. *AMIA Summits Transl Sci Proc* 2024;2024:459. PMC11141850 38827061

[ref101] Zeng Z, Wu T, Lv M et al. Impact of mobile health and telehealth technology on medication adherence of stroke patients: a systematic review and meta-analysis of randomized controlled trials. *Int J Clin Pharm* 2022;44:4–14. 10.1007/s11096-021-01351-x 34800254

[ref102] Zhang Y . Measuring and applying digital literacy: implications for access for the elderly in rural China. *Educ Inf Technol* 2023;28:9509–28. 10.1007/s10639-022-11448-z

[ref104] Zhuang Q, Chen F, Wang T. Effectiveness of short message service intervention to improve glycated hemoglobin control and medication adherence in type-2 diabetes: a meta-analysis of prospective studies. *Prim Care Diabetes* 2020;14:356–63. 10.1016/j.pcd.2019.09.007 31653588

