# Supplementary material for: The impact of patient-facing digital health interventions on patient safety outcomes: a meta-analysis of meta-analyses
Source: Oxf Open Digit Health. 2026 Aug 29;4:oqag031. doi: 10.1093/oodh/oqag031 (PMC13587088; doi:10.1093/oodh/oqag031)
Supplement: Supplementary file 1 [file supplementary_materials_oqag031.docx]

**Title:**

The impact of patient-facing digital health interventions on patient safety outcomes: a meta-analysis of meta-analyses

**Short running title:** Digital health interventions and patient safety

**List of authors:** Goran ERFANI, Jemma McCREADY, Beckie GIBSON, Alison STEVEN, John UNSWORTH, Heidi SIIRA, Erika JARVA, Kristina MIKKONEN, Marco TOMIETTO*.

**Authors:**

1. Goran ERFANI, PhD, Research Fellow, School of Healthcare and Nursing Sciences, Faculty of Health and Wellbeing, Northumbria University, Newcastle upon Tyne, United Kingdom; email: [goran.erfani@northumbria.ac.uk](mailto:goran.erfani@northumbria.ac.uk); ORCID: https://orcid.org/0000-0002-8135-2846; X: @GoranErfani
2. Jemma McCREADY, PhD, MSc, BSc (Hons), Research Assistant, Department of Social Work, Education and Community Wellbeing, Faculty of Health and Life Sciences, Northumbria University, Newcastle upon Tyne, United Kingdom. [j.mccready@northumbria.ac.uk](mailto:j.mccready@northumbria.ac.uk); ORCID: https://orcid.org/0000-0002-9997-6956; X: @JemmaMcC90
3. Beckie GIBSON, MSc, BSc (Hons), Research Assistant, Psychology Department, Northumbria University, Newcastle upon Tyne, United Kingdom. beckie2.gibson@northumbria.ac.uk;
4. Alison STEVEN, PhD, MSc, BSc (Hons), RN, Professor, School of Healthcare and Nursing Sciences, Faculty of Health and Wellbeing, Northumbria University, Newcastle upon Tyne, United Kingdom; alison.steven@northumbria.ac.uk; ORCID:  [https://orcid.org/0000-0003-4811-1706;](https://orcid.org/0000-0002-7642-1448;) X: @AlisonSteven3
5. John UNSWORTH, PhD, LLM, MSc, BSc (Hons), BA, RN, PGCE, NTF, PFHEA, FEANS, FFNMRCSI, Professor, School of Healthcare and Nursing Sciences, Faculty of Health and Wellbeing, Northumbria University, Newcastle upon Tyne, United Kingdom. john.unsworth@northumbria.ac.uk; ORCID: https://orcid.org/0000-0002-4150-6513; X: @johnunsworth10.
6. Heidi SIIRA, PhD, Post-Doctoral researcher, PT, Research Unit of Health Sciences and Technology, University of Oulu, Oulu. heidi.siira@oulu.fi; ORCID: https://orcid.org/0000-0001-8707-0164; X: @SiiraHeidi
7. Erika JARVA, PhD, Post-Doctoral Researcher, PT, Research Unit of Health Sciences and Technology, Faculty of Medicine, University of Oulu, Finland; erika.jarva@oulu.fi; ORCID: https://orcid.org/0000-0001-6860-4319; X: @ErikaJarva
8. Kristina MIKKONEN, PhD, RN, Professor, Research Unit of Health Sciences and Technology, University of Oulu, Oulu, Medical Research Center Oulu, Oulu University Hospital and University of Oulu, Oulu, Finland; Department of Nursing, Midwifery and Health, Faculty of Health and Life Sciences, Northumbria University, Newcastle upon Tyne, United Kingdom; [kristina.mikkonen@oulu.fi](mailto:kristina.mikkonen@oulu.fi); ORCID: <https://orcid.org/0000-0002-4355-3428>; X: @Kristinamikkon;
9. Marco TOMIETTO, PhD, MSc, RN, Professor of Nursing, School of Healthcare and Nursing Sciences, Faculty of Health and Wellbeing, Northumbria University, Newcastle upon Tyne, United Kingdom; Centre for Health and Social Equity (CHASE), Northumbria University, Newcastle upon Tyne, United Kingdom; Adjunct Professor, Research Unit of Health Science and Technology, University of Oulu, Oulu (Finland);
   marco.tomietto@northumbria.ac.uk; ORCID: https://orcid.org/0000-0002-3813-1490; X: @marco_tomietto.

**Corresponding Author:** Marco Tomietto, Coach Lane Campus East Building, School of Healthcare and Nursing Sciences, Faculty of Health and Wellbeing, Northumbria University, Newcastle upon Tyne, NE7 7TR, United Kingdom.

e-mail: marco.tomietto@northumbria.ac.uk; telephone number: +44 (0191) 215 6060

**Conflict of Interest Statement:** The authors have no conflicts of interest to declare

**Funding statement:** This study is funded by Northumbria University.

**Data availability statement:** Data supporting this study are available as supplementary files.

**Ethical Approval:** not applicable

**Declaration of Generative AI and AI-assisted technologies in the writing process**

During the preparation of this work the author(s) used an organisational-protected licence of Claude in order to edit the English language and enhance sentence structure in the text excerpts. After using this tool/service, the author(s) reviewed and edited the content as needed and take(s) full responsibility for the content of the publication.

**CRediT statement and authors’ contributions:**
Study design: MT, JU, AS, JM.
Data collection: JM, GE, BG
Data analysis: GE, MT, JM
Study supervision: MT, JU, AS
Manuscript writing: GE, BG, MT
Critical revisions for important intellectual content: MT, KM, JU, AS, HS, EJ

All authors meet the journal’s criteria for authorship, and all authors have drafted and reviewed the current manuscript and agree with its submission.

Appendix I

Table S1. Summary of the key characteristics of the included reviews and risk of bias assessment

| **No.** | **First Author & Year of Publication** | **Intervention Type** | **Health Condition** | **Outcomes Assessed** | **Number of Participants** | **Number of Primary Studies Pooled** | **Quality of Included Review based on JBI Critical Appraisal Checklist** |
| --- | --- | --- | --- | --- | --- | --- | --- |
| 1 | Aardoom et al., 2020 | eHealth | Obstructive Sleep Apnea Patients | Medication adherence | 5429 | 18 | Strong |
| 2 | Akinosun et al., 2021 | Digital tech | Cardiovascular Disease | Medication adherence | 2710 | 11 | Low |
| 3 | Al-Arkee et al. 2021 | mHealth | Cardiovascular disease | Medication adherence | 442 | 6 | Strong |
| 4 | Amankwaa et al., 2018 | mHealth | HIV | Medication adherence | 1468 | 8 | Strong |
| 5 | Ambrens et al., 2022 | eHealth | Community dwelling older adults | Falls | 823 | 5 | Strong |
| 6 | Armitage et al., 2020 | mHealth | No specific health condition | Medication adherence | 988 | 9 | Moderate |
| 7 | Belete et al., 2023 | mHealth | Type 2 Diabetes | Medication adherence | 1121 | 9 | Strong |
| 8 | Boima et al., 2024 | mHealth | Hypertension | Medication adherence (dichot.) | 4060 | 5 | Moderate |
|  |  |  |  | Medication adherence (cont.) | 1206 | 8 |  |
| 9 | Braver et al., 2023 | mHealth | Coronary Artery Disease | Adverse events | 588 | 4 | Strong |
|  |  |  |  | Healthcare utilisation | 199 | 2 |  |
|  |  |  |  | Mortality | 2711 | 8 |  |
|  |  |  |  | Readdmistion | 1514 | 10 |  |
| 10 | Cao et al., 2024 | mHealth | Stroke | Medication adherence | 257 | 2 | Strong |
| 11 | Chan et al. 2021 | e-interventions | Community Dwelling Older Adults | Fals (dichot.) | 2500 | 11 | Strong |
|  |  |  |  | Falls (cont.) | 429 | 7 |  |
| 12 | Chilala et al., 2023 | Telemedicine | Cardiometabolic Conditions | Healthcare utilisation | 4182 | 2 | Strong |
|  |  |  |  | Mortality | 1071 | 7 |  |
|  |  |  |  | Medication adherence (dichot.) | 4823 | 17 |  |
|  |  |  |  | Medication adherence (cont.) | 20271 | 24 |  |
|  |  |  |  | Readdmistion | 5402 | 6 |  |
| 13 | Choi et al., 2023 | mHealth | Stroke | Medication adherence (dichot.) | 1246 | 3 | Strong |
|  |  |  |  | Medication adherence (cont.) | 668 | 7 |  |
| 14 | Chua et al., 2022 | Telehealth | Helicobacter pylori eradication | Adverse events | 828 | 5 | Strong |
|  |  |  |  | Medication adherence | 685 | 7 |  |
| 15 | Clark et al., 2007 | Telemonitoring | Chronic Heart Failure | Healthcare utilisation | 441 | 2 | Low |
|  |  |  |  | Mortality | 807 | 5 |  |
| 16 | Clarke et al., 2011 | Telemonitoring | Congestive Heart Failure | Healthcare utilisation | 1951 | 6 | Very low |
|  |  |  |  | Mortality | 2171 | 10 |  |
| 17 | Dawes et al., 2021 | mHealth | Post-operative Patients | Healthcare utilisation | 582 | 4 | Very low |
|  |  |  |  | Readdmistion | 848 | 6 |  |
| 18 | Deng et al., 2022 | Telehealth | Atherosclerotic Cardiovascular Disease | Adverse events | 659 | 3 | Moderate |
|  |  |  |  | Healthcare utilisation | 1397 | 3 |  |
| 19 | Drews et al., 2021 | Telemonitoring | Patients with Decompensated Heart Failure | Healthcare utilisation | 4291 | 9 | Low |
|  |  |  |  | Mortality | 2521 | 11 |  |
| 20 | Esmaeili et al., 2023 | Telehealth | HIV | Medication adherence (dichot.) | 975 | 5 | Very low |
|  |  |  |  | Medication adherence (cont.) | 916 | 5 |  |
| 21 | Farmer et al., 2016 | mHealth | Type 2 Diabetes | Medication adherence | 1131 | 8 | Strong |
| 22 | Finitsis et al., 2014 | mHealth | HIV | Medication adherence | 1687 | 9 | Strong |
| 23 | Gandhi et al., 2017 | mHealth | Cardiovascular Disease | Medication adherence | 1355 | 9 | Strong |
|  |  |  |  | Mortality | 228 | 2 |  |
|  |  |  |  | Readdmistion | 2436 | 5 |  |
| 24 | Gordon et al., 2023 | Telehealth | Inflammatory Bowel Disease | Healthcare utilisation | 86 | 1 | Strong |
|  |  |  |  | Medication adherence (dichot.) | 220 | 5 |  |
|  |  |  |  | Medication adherence (cont.) | 671 | 1 |  |
| 25 | Gurcay et al., 2024 | Telehealth | Type 2 Diabetes | Medication adherence | 5644 | 18 | Low |
| 26 | Huang et al., 2015 | Telehealth | Coronary Heart Disease | Mortality | 1303 | 4 | Low |
| 27 | Hwang & Chang, 2023 | Nurse-led digital health interventions (mHealh, eHealth, Telehealth) | Hypertension | Medication adherence | 647 | 6 | Moderate |
| 28 | Iqbal et al., 2021 | Telemonitoring | Medical and surgical cohorts | Healthcare utilisation | 6972 | 21 | Low |
|  |  |  |  | Mortality | NA | 12 |  |
| 29 | Jeminiwa et al., 2019 | eHealth | Asthma | Medication adherence | 19277 | 10 | Low |
| 30 | Jin et al., 2019 | Telehealth | Coronary Heart Disease | Mortality | 5075 | 14 | Moderate |
| 31 | Kashgary et al., 2017 | mHealth | No specific health condition | Medication adherence | 3355 | 13 | Very low |
| 32 | Kassavou et al., 2018 | Telehealth | Cardio-metabolic conditions | Medication adherence | 38671 | 17 | Moderate |
| 33 | Kassavou et al., 2022 | mHealth | Hypertension | Medication adherence (dichot.) | 6428 | 4 | Strong |
|  |  |  |  | Medication adherence (cont.) | 688 | 4 |  |
| 34 | Klersy et al., 2009 | Telemonitoring | Heart Failure | Healthcare utilisation | 4122 | 11 | Very low |
|  |  |  |  | Mortality | 8058 | 23 |  |
| 35 | Kotb et al., 2014 | Telemedicine | Coronary Artery Disease | Healthcare utilisation | 706 | 4 | Moderate |
|  |  |  |  | Mortality | 2937 | 11 |  |
| 36 | Labarca et al., 2021 | Telemedicine | Obstructive Sleep Apnea Patients | Medication adherence | 3039 | 16 | Strong |
| 37 | Lee et al., 2020 | eHealth | Organ Transplant patients | Medication adherence | 454 | 5 | Low |
| 38 | Liu et al., 2022 | eHealth | Heart Failure | Medication adherence | 1051 | 3 | Strong |
|  |  |  |  | Mortality | 6610 | 15 |  |
|  |  |  |  | Readdmistion | 4310 | 16 |  |
| 39 | Ma et al., 2019 | eHealth | Hypertension | Medication adherence | 906 | 4 | Moderate |
| 40 | Mayer & Fontelo, 2017 | mHealth | HIV | Medication adherence | 3575 | 20 | Very low |
| 41 | Mehra et al., 2021 | mHealth | HIV | Medication adherence | 558 | 3 | Low |
| 42 | Menezes Junior et al., 2023 | Telehealth | Patients with cardiac implantable electronic devices | Healthcare utilisation (cont.) | 1502 | 5 | Strong |
|  |  |  |  | Healthcare utilisation (dichot.) | 1548 | 5 |  |
|  |  |  |  | Mortality | 3006 | 6 |  |
| 43 | Mikulski et al., 2022 | mHealth | Hypertension | Medication adherence | 627 | 6 | Moderate |
| 44 | Pang et al., 2022 | Telehealth | Inflammatory Bowel Disease | Healthcare utilisation | 1479 | 7 | Strong |
|  |  |  |  | Medication adherence | 819 | 3 |  |
|  |  |  |  | Relapse rate | 774 | 5 |  |
| 45 | Polisena et al., 2010 | Telemonitoring | Congestive Heart Failure | Healthcare utilisation | 891 | 4 | Low |
|  |  |  |  | Mortality | 1304 | 6 |  |
| 46 | Ramachandran et al., 2022 | Telerehabilitation | Coronary Heart Disease | Mortality | 1465 | 4 | Moderate |
| 47 | Rohde et al., 2021 | eHealth | Chronic Gastrointestinal Illnesses | Healthcare utilisation | 1165 | 5 | Very low |
|  |  |  |  | Medication adherence | 827 | 4 |  |
| 48 | Saragih et al., 2023 | Telehealth | Cardiovascular disease | Medication adherence | 1280 | 5 | Strong |
| 49 | Saragih et al., 2024 | Telehealth | HIV/AIDS | Medication adherence | 861 | 3 | Strong |
| 50 | Shah et al., 2019 | mHealth | HIV | Medication adherence | 146 | 3 | Strong |
| 51 | Solis-Navarro et al. 2022 | Digital health interventions | Community Dwelling Older Adult | Falls | 2131 | 7 | Moderate |
| 52 | Son et al., 2020 | mHealth | Chronic Heart Failure | Healthcare utilisation | 458 | 2 | Strong |
|  |  |  |  | Mortality | 907 | 3 |  |
| 53 | Song et al., 2021 | eHealth | Chronic Obstructive Pulmonary Disease and Asthma | Healthcare utilisation | 609 | 6 | Moderate |
| 54 | Stevenson et al., 2019 | eHealth | Chronic Kidney Disease | Mortality | 2906 | 3 | Strong |
| 55 | Su et al., 2020 | eHealth | Coronary Heart Disease | Readdmistion | 433 | 3 | Moderate |
| 56 | Sul et al., 2020 | Telemonitoring | Chronic Obstructive Pulmonary Disease | Healthcare utilisation | 688 |  | Very low |
|  |  |  |  | Mortality | 919 | 7 |  |
| 57 | Sun et al., 2023 | mHealth | HIV | Medication adherence | 2110 | 7 | Moderate |
| 58 | Sun et al., 2024 | Telehealth | Breast cancer | Medication adherence (dichot.) | 1274 | 5 | Strong |
|  |  |  |  | Medication adherence (cont.) | 701 | 5 |  |
| 59 | Tam et al., 2022 | mHealth | Hypertension | Medication adherence | 693 | 3 | Moderate |
| 60 | Tang et al., 2020 | eHealth | Solid Organ Transplant Recipients | Adverse events | 442 | 3 | Strong |
|  |  |  |  | Medication adherence | 306 | 5 |  |
|  |  |  |  | Mortality | 406 | 2 |  |
|  |  |  |  | Healthcare utilisation (cont.) | 132 | 3 |  |
|  |  |  |  | Healthcare utilisation (dichot.) | 395 | 4 |  |
| 61 | Thakkar et al., 2016 | eHealth | Chronic Diseases | Medication adherence | 2742 | 16 | Strong |
| 62 | ting Zhou et al., 2022 | mmApps | Chronic Diseases | Adverse events | 579 | 3 | Very low |
|  |  |  |  | Medication adherence | 466 | 2 |  |
| 63 | Tsuge et al., 2023 | Telerehabilitation | Hip fracture surgery patients | Adverse events | 364 | 6 | Moderate |
| 64 | Wang et al., 2019 | eHealth | HIV | Medication adherence | 3937 | 21 | Strong |
| 65 | Xiang et al., 2013 | Telehealth | Chronic Heart Failure | Healthcare utilisation | 5675 | 23 | Very low |
|  |  |  |  | Mortality | 6162 | 27 |  |
| 66 | Xu & Long, 2020 | mHealth | Hypertension | Medication adherence | 1109 | 4 | Moderate |
| 67 | Yang et al., 2018 | mHealth | Chronic Obstructive Pulmonary Disease | Healthcare utilisation | 337 | 8 | Moderate |
| 68 | Yang et al., 2023 | Hybrid Comprehensive Telerehabilitation (HCTR) | Cardiovascular disease | Mortality | 1081 | 3 | Moderate |
|  |  |  |  | Readdmistion | 983 | 3 |  |
| 69 | Yun et al., 2018 | Telemonitoring | Heart failure | Healthcare utilisation | 6982 | 17 | Moderate |
|  |  |  |  | Mortality | 7457 | 24 |  |
| 70 | Zeng et al., 2022 | mHealth | Stroke | Medication adherence | 498 | 4 | Moderate |
| 71 | Zhu et al., 2019 | Telemonitoring | Heart failure | Healthcare utilisation | 5543 | 17 | Moderate |
|  |  |  |  | Mortality | 4963 | 15 |  |
| 72 | Zhuang et al., 2020 | SMS messaging intervention | Type 2 Diabetes | Medication adherence | 164 | 3 | Very low |

Appendix II

Figure S1. Funnel plot of effect on medication adherence (SMD)

Figure S2. Funnel plot of effect on medication adherence (RRs)

Figure S3. Funnel plot of effect on healthcare utilisation (SMD)

Figure S4. Funnel plot of effect on healthcare utilisation (RRs)

Figure S5. Funnel plot of effect on falls (SMD)

Figure S6. Funnel plot of effect on falls (RRs)

Figure S7. Funnel plot of effect on adverse events (RRs)

Figure S8. Funnel plot of effect on mortality (RRs)

Figure S9. Funnel plot of effect on relapse rate (RRs)

Figure S10. Funnel plot of effect on readmission (RRs)

Appendix III

Table S2. Included studies

| **No.** | **Citation** | **Article Title** | **DOI** |
| --- | --- | --- | --- |
| 1 | Aardoom et al., 2020 | Effectiveness of eHealth Interventions in Improving Treatment Adherence for Adults With Obstructive Sleep Apnea: Meta-Analytic Review. | 10.2196/16972 |
| 2 | Akinosun et al., 2021 | Digital Technology Interventions for Risk Factor Modification in Patients With Cardiovascular Disease: Systematic Review and Meta-analysis | 10.2196/21061 |
| 3 | Al-Arkee et al. 2021 | Mobile Apps to Improve Medication Adherence in Cardiovascular Disease: Systematic Review and Meta-analysis. | 10.2196/24190 |
| 4 | Amankwaa et al., 2018 | Effectiveness of short message services and voice call interventions for antiretroviral therapy adherence and other outcomes: A systematic review and meta-analysis. | 10.1371/journal.pone.0204091 |
| 5 | Ambrens et al., 2022 | Effect of eHealth-delivered exercise programmes on balance in people aged 65 years and over living in the community: a systematic review and meta-analysis of randomised controlled trials | 10.1136/bmjopen-2021-051377 |
| 6 | Armitage et al., 2020 | Do mobile device apps designed to support medication adherence demonstrate efficacy? A systematic review of randomised controlled trials, with meta-analysis | 10.1136/bmjopen-2019-032045 |
| 7 | Belete et al., 2023 | What is the effect of mobile phone text message reminders on medication adherence among adult type 2 diabetes mellitus patients: a systematic review and meta-analysis of randomized controlled trials. | 10.1186/s12902-023-01268-8 |
| 8 | Boima et al., 2024 | Effectiveness of digital health interventions on blood pressure control, lifestyle behaviours and adherence to medication in patients with hypertension in low-income and middle-income countries: a systematic review and meta-analysis of randomised controll | 10.1016/j.eclinm.2024.102432 |
| 9 | Braver et al., 2023 | Digital health programs to reduce readmissions in coronary artery disease: a systematic review and meta-analysis. | 10.1016/j.jacadv.2023.100591 |
| 10 | Cao et al., 2024 | Effectiveness of mobile application interventions for stroke survivors: systematic review and meta-analysis | 10.1186/s12911-023-02391-1 |
| 11 | Chan et al. 2021 | The effectiveness of e-interventions on fall, neuromuscular functions and quality of life in community-dwelling older adults: A systematic review and meta-analysis. | 10.1016/j.ijnurstu.2020.103784 |
| 12 | Chilala et al., 2023 | Evaluating the Effectiveness of Remote Behavioral Interventions Facilitated by Health Care Providers at Improving Medication Adherence in Cardiometabolic Conditions: A Systematic Review and Meta-Analysis | 10.1093/abm/kaac037 |
| 13 | Choi et al., 2023 | Effectiveness of Remote Interventions to Improve Medication Adherence in Patients after Stroke: A Systematic Literature Review and Meta-Analysis | 10.3390/bs13030246 |
| 14 | Chua et al., 2022 | Does technology-enhanced communication improve Helicobacter pylori eradication outcomes?-A meta-analysis | 10.1111/hel.12890 |
| 15 | Clark et al., 2007 | Telemonitoring or structured telephone support programmes for patients with chronic heart failure: systematic review and meta-analysis | 10.1136/bmj.39156.536968.55 |
| 16 | Clarke et al., 2011 | Systematic review of studies on telemonitoring of patients with congestive heart failure: a meta-analysis. | 10.1258/jtt.2010.100113 |
| 17 | Dawes et al., 2021 | Mobile health technology for remote home monitoring after surgery: a meta-analysis | 10.1093/bjs/znab323 |
| 18 | Deng et al., 2022 | The effect of telemedicine on secondary prevention of atherosclerotic cardiovascular disease: A systematic review and meta-analysis | 10.3389/fcvm.2022.1020744 |
| 19 | Drews et al., 2021 | Non‐invasive home telemonitoring in patients with decompensated heart failure: a systematic review and meta‐analysis. | 10.1002/ehf2.13475 |
| 20 | Esmaeili et al., 2023 | Does telehealth affect the adherence to ART among patients with HIV? A systematic review and meta-analysis | 10.1186/s12879-023-08119-w |
| 21 | Farmer et al., 2016 | Effects of interventions promoting monitoring of medication use and brief messaging on medication adherence for people with type 2 diabetes: a systematic review of randomized trials. | 10.1111/dme.12987 |
| 22 | Finitsis et al., 2014 | Text message intervention designs to promote adherence to antiretroviral therapy (ART): a meta-analysis of randomized controlled trials. | 10.1371/journal.pone.0088166 |
| 23 | Gandhi et al., 2017 | Effect of mobile health interventions on the secondary prevention of cardiovascular disease: systematic review and meta-analysis. | 10.1016/j.cjca.2016.08.017 |
| 24 | Gordon et al., 2023 | Remote care through telehealth for people with inflammatory bowel disease | 10.1002/14651858.CD014821.pub2 |
| 25 | Gurcay et al., 2024 | The Effectiveness of Telehealth Interventions on Medication Adherence Among Patients with Type 2 Diabetes: A Meta-Analysis | 10.1089/tmj.2023.0088 |
| 26 | Huang et al., 2015 | Distance management of inflammatory bowel disease: systematic review and meta-analysis | 10.3748/wjg.v20.i3.829 |
| 27 | Hwang & Chang, 2023 | The effect of nurse-led digital health interventions on blood pressure control for people with hypertension: A systematic review and meta-analysis. | 10.1111/jnu.12882 |
| 28 | Iqbal et al., 2021 | Clinical outcomes of digital sensor alerting systems in remote monitoring: a systematic review and meta-analysis | 10.1038/s41746-020-00378-0 |
| 29 | Jeminiwa et al., 2019 | Impact of eHealth on medication adherence among patients with asthma: A systematic review and meta-analysis | 10.1016/j.rmed.2019.02.011 |
| 30 | Jin et al., 2019 | Telehealth interventions for the secondary prevention of coronary heart disease: a systematic review and meta-analysis. | 10.1177/1474515119826510 |
| 31 | Kashgary et al., 2017 | The role of mobile devices in doctor-patient communication: a systematic review and meta-analysis | 10.1177/1357633X16661604 |
| 32 | Kassavou et al., 2018 | Automated telecommunication interventions to promote adherence to cardio-metabolic medications: meta-analysis of effectiveness and meta-regression of behaviour change techniques. | 10.1080/17437199.2017.1365617 |
| 33 | Kassavou et al., 2022 | The Association Between Smartphone App-Based Self-monitoring of Hypertension-Related Behaviors and Reductions in High Blood Pressure: Systematic Review and Meta-analysis | 10.2196/34767 |
| 34 | Klersy et al., 2009 | A meta-analysis of remote monitoring of heart failure patients. | 10.1016/j.jacc.2009.08.017 |
| 35 | Kotb et al., 2014 | The effect of telephone support interventions on coronary artery disease (CAD) patient outcomes during cardiac rehabilitation: a systematic review and meta-analysis. | 10.1371/journal.pone.0096581 |
| 36 | Labarca et al., 2021 | Telemedicine interventions for CPAP adherence in obstructive sleep apnea patients: Systematic review and meta-analysis. | 10.1016/j.smrv.2021.101543 |
| 37 | Lee et al., 2020 | Effectiveness of eHealth interventions for improving medication adherence of organ transplant patients: A systematic review and meta-analysis | 10.1371/journal.pone.0241857 |
| 38 | Liu et al., 2022 | Effectiveness of eHealth Self-management Interventions in Patients With Heart Failure: Systematic Review and Meta-analysis | 10.2196/38697 |
| 39 | Ma et al., 2019 | The effectiveness of electronic health interventions on blood pressure control, self-care behavioural outcomes and psychosocial well-being in patients with hypertension: A systematic review and meta-analysis. | 10.1016/j.ijnurstu.2018.11.007 |
| 40 | Mayer & Fontelo, 2017 | Meta-analysis on the effect of text message reminders for HIV-related compliance | 10.1080/09540121.2016.1214674 |
| 41 | Mehra et al., 2021 | Effectiveness of mobile phone text message reminder interventions to improve adherence to antiretroviral therapy among adolescents living with HIV: A systematic review and meta-analysis. | 10.1371/journal.pone.0254890 |
| 42 | Menezes Junior et al., 2023 | Long-Term Remote vs. Conventional Monitoring of Pacemakers: Systematic Review and Meta-Analysis of Randomized Controlled Trials | 10.1007/s11886-023-01963-x |
| 43 | Mikulski et al., 2022 | Mobile Health Applications and Medication Adherence of Patients With Hypertension: A Systematic Review and Meta-Analysis. | 10.1016/j.amepre.2021.11.003 |
| 44 | Pang et al., 2022 | Role of Telemedicine in Inflammatory Bowel Disease: Systematic Review and Meta-analysis of Randomized Controlled Trials | 10.2196/28978 |
| 45 | Polisena et al., 2010 | Home telemonitoring for congestive heart failure: a systematic review and meta-analysis. | 10.1258/jtt.2009.090406 |
| 46 | Ramachandran et al., 2022 | Effectiveness of home-based cardiac telerehabilitation as an alternative to Phase 2 cardiac rehabilitation of coronary heart disease: a systematic review and meta-analysis | 10.1093/eurjpc/zwab106 |
| 47 | Rohde et al., 2021 | Impact of eHealth technologies on patient outcomes: a meta-analysis of chronic gastrointestinal illness interventions | 10.1093/tbm/ibz166 |
| 48 | Saragih et al., 2023 | Effects of telehealth-assisted interventions for secondary prevention of cardiovascular disease: A systematic review and meta-analysis. | 10.1111/jocn.16452 |
| 49 | Saragih et al., 2024 | Effects of telehealth-assisted interventions among people living with HIV/AIDS: A systematic review and meta-analysis of randomized controlled studies | 10.1177/1357633X211070726 |
| 50 | Shah et al., 2019 | A systematic review and meta-analysis in the effectiveness of mobile phone interventions used to improve adherence to antiretroviral therapy in HIV infection. | 10.1186/s12889-019-6899-6 |
| 51 | Solis-Navarro et al. 2022 | Effectiveness of home-based exercise delivered by digital health in older adults: a systematic review and meta-analysis. | 10.1093/ageing/afac243 |
| 52 | Son et al., 2020 | Effectiveness of mobile phone-based interventions for improving health outcomes in patients with chronic heart failure: a systematic review and meta-analysis. | 10.3390/ijerph17051749 |
| 53 | Song et al., 2021 | Blended Self-Management Interventions to Reduce Disease Burden in Patients With Chronic Obstructive Pulmonary Disease and Asthma: Systematic Review and Meta-analysis. | 10.2196/24602 |
| 54 | Stevenson et al., 2019 | eHealth interventions for people with chronic kidney disease | 10.1002/14651858.CD012379.pub2 |
| 55 | Su et al., 2020 | Effect of eHealth cardiac rehabilitation on health outcomes of coronary heart disease patients: A systematic review and meta‐analysis. | 10.1111/jan.14272 |
| 56 | Sul et al., 2020 | Effectiveness of telemonitoring versus usual care for chronic obstructive pulmonary disease: A systematic review and meta-analysis. | 10.1177/1357633X18811757 |
| 57 | Sun et al., 2023 | Effectiveness of mHealth on Adherence to Antiretroviral Therapy in Patients Living With HIV: Meta-analysis of Randomized Controlled Trials | 10.2196/42799 |
| 58 | Sun et al., 2024 | Effect of telehealth interventions on adherence to endocrine therapy among patients with breast cancer: a systematic review and meta-analysis | 10.1007/s00520-024-08339-z |
| 59 | Tam et al., 2022 | Integration of text messaging interventions into hypertension management among older adults: A systematic review and meta-analysis | 10.1111/wvn.12549 |
| 60 | Tang et al., 2020 | eHealth Interventions for Solid Organ Transplant Recipients: A Systematic Review and Meta-analysis of Randomized Controlled Trials | 10.1097/tp.0000000000003294 |
| 61 | Thakkar et al., 2016 | Mobile telephone text messaging for medication adherence in chronic disease: a meta-analysis. | 10.1001/jamainternmed.2015.7667 |
| 62 | ting Zhou et al., 2022 | Effectiveness of Mobile medical apps in ensuring medication safety among patients with chronic diseases: systematic review and meta-analysis | 10.2196/39819 |
| 63 | Tsuge et al., 2023 | Efficacy of telerehabilitation for patients after hip fracture surgery: A systematic review and meta-analysis | 10.1177/1357633X231181632 |
| 64 | Wang et al., 2019 | Electronic Health Interventions to Improve Adherence to Antiretroviral Therapy in People Living With HIV: Systematic Review and Meta-Analysis | 10.2196/14404 |
| 65 | Xiang et al., 2013 | Meta-analysis and meta-regression of telehealth programmes for patients with chronic heart failure | 10.1177/1357633X13495490 |
| 66 | Xu & Long, 2020 | The effect of smartphone app–based interventions for patients with hypertension: systematic review and meta-analysis. | 10.2196/21759 |
| 67 | Yang et al., 2018 | Mobile health applications in self-management of patients with chronic obstructive pulmonary disease: a systematic review and meta-analysis of their efficacy. | 10.1186/s12890-018-0671-z |
| 68 | Yang et al., 2023 | Efficacy and Safety of Hybrid Comprehensive Telerehabilitation (HCTR) for Cardiac Rehabilitation in Patients with Cardiovascular Disease: A Systematic Review and Meta-Analysis of Randomized Controlled Trials. | 10.1155/2023/5147805 |
| 69 | Yun et al., 2018 | Comparative effectiveness of telemonitoring versus usual care for heart failure: a systematic review and meta-analysis. | 10.1016/j.cardfail.2017.09.006 |
| 70 | Zeng et al., 2022 | Impact of mobile health and telehealth technology on medication adherence of stroke patients: a systematic review and meta-analysis of randomized controlled trials | 10.1007/s11096-021-01351-x |
| 71 | Zhu et al., 2019 | Effectiveness of telemedicine systems for adults with heart failure: a meta-analysis of randomized controlled trials. | 10.1007/s10741-019-09801-5 |
| 72 | Zhuang et al., 2020 | Effectiveness of short message service intervention to improve glycated hemoglobin control and medication adherence in type-2 diabetes: A meta-analysis of prospective studies. | 10.1016/j.pcd.2019.09.007 |

Appendix IV

Table S3. Mapping of the nine patient safety outcomes assessed in this meta-analysis of meta-analyses onto Donabedian's Structure-Process-Outcome framework (Donabedian, 1988), presented alongside pooled effect estimates, number of contributing meta-analyses, and overall finding for each outcome.

| **Outcome** | **Donabedian category** | **Metric (measure)** | **Pooled effect (95% CI)** | **No. of meta-analyses** | **Overall finding** |
| --- | --- | --- | --- | --- | --- |
| Medication adherence | Process | SMD | 0.33 (0.22 to 0.44), P < 0.01 | 29 | Positive |
| Medication adherence | Process | RR | 1.22 (1.15 to 1.29), P < 0.01 | 26 | Positive |
| Healthcare utilisation | Process | SMD | -0.05 (-0.35 to 0.24), P = 0.74 | 7 | Inconclusive |
| Healthcare utilisation | Process | RR | 0.90 (0.85 to 0.94), P < 0.01 | 18 | Positive |
| Falls | Outcome | SMD | 0.30 (0.13 to 0.46), P < 0.01 | 2 | Positive |
| Falls | Outcome | RR | 0.82 (0.73 to 0.92), P < 0.01 | 2 | Positive |
| Adverse events | Outcome | RR | 0.94 (0.66 to 1.34), P = 0.73 | 6 | Inconclusive |
| Medication errors | Outcome | — | Insufficient data for meta-analysis (1 study) | 0 | Not assessable |
| Mortality | Outcome | RR | 0.84 (0.78 to 0.90), P < 0.01 | 22 | Positive |
| Relapse rate | Outcome | RR | 1.00 (0.87 to 1.16), P = 0.97 | 2 | Inconclusive |
| Readmissions | Outcome | RR | 0.90 (0.81 to 0.98), P < 0.01 | 7 | Positive |

Note: No outcomes in this review corresponded to the Structure domain of the Donabedian framework (e.g., staffing levels, facility characteristics, technology infrastructure), as this domain falls outside the scope of patient-facing intervention effects on individual patient safety outcomes. SMD = standardised mean difference; RR = risk ratio. “Inconclusive” denotes a pooled 95% confidence interval crossing the null value (0 for SMD, 1 for RR). “Not assessable” denotes outcomes with insufficient primary meta-analyses (fewer than two) to support pooling.
